# Temporal dynamics of ecosystem, inherent, and underlying water use efficiencies of forests, grasslands, and croplands and their responses to climate change

**DOI:** 10.1186/s13021-023-00232-2

**Published:** 2023-07-14

**Authors:** Wei Chen, Shuguang Liu, Shuqing Zhao, Yu Zhu, Shuailong Feng, Zhao Wang, Yiping Wu, Jingfeng Xiao, Wenping Yuan, Wende Yan, Hui Ju, Qinyi Wang

**Affiliations:** 1 College of Life Science and Technology, Central South University of Forestry and Technology, Changsha, 410004 China; 2 National Engineering Laboratory for Applied Technology of Forestry and Ecology in South China, Central South University of Forestry and Technology, Changsha, 410004 China; 3 College of Urban and Environmental Sciences, Key Laboratory for Earth Surface Processes of the Ministry of Education, Peking University, Beijing, 100871 China; 4 Department of Earth and Environmental Science, Xi’an Jiaotong University, Xi’an, 710049 Shaanxi China; 5 Earth Systems Research Center, Institute for the Study of Earth, Oceans, and Space, University of New Hampshire, Durham, NH 03824 USA; 6 School of Atmospheric Sciences, Southern Marine Science and Engineering Guangdong Laboratory (Zhuhai), Sun Yat-sen University, Zhuhai, 519082 Guangdong China; 7 Institute of Environment and Sustainable Development in Agriculture, Chinese Academy of Agricultural Science, Beijing, 100081 China; 8 Key Laboratory of Regional Ecology and Environmental Change, School of Geography and Information Engineering, China University of Geosciences, Wuhan, 430074 China

**Keywords:** Ecosystem level, Water use efficiency, FLUXNET, Changing trend, Driving factors

## Abstract

**Background:**

Understanding temporal trends and varying responses of water use efficiency (WUE) to environmental changes of diverse ecosystems is key to predicting vegetation growth. WUE dynamics of major ecosystem types (e.g., forest, grassland and cropland) have been studied using various WUE definitions/metrics, but a comparative study on WUE dynamics and their driving forces among different ecosystem types using multiple WUE metrics is lacking. We used eddy covariance measurements for 42 FLUXNET2015 sites (396 site years) from 1997 to 2014, as well as three commonly used WUE metrics (i.e., ecosystem, inherent, and underlying WUE) to investigate the commonalities and differences in WUE trends and driving factors among deciduous broadleaf forests (DBFs), evergreen needleleaf forests (ENFs), grasslands, and croplands.

**Results:**

Our results showed that the temporal trends of WUE were not statistically significant at 73.8% of the forest, grassland and cropland sites, and none of the three WUE metrics exhibited better performance than the others in quantifying WUE. Meanwhile, the trends observed for the three WUE metrics were not significantly different among forest, grassland and cropland ecosystems. In addition, WUE was mainly driven by atmospheric carbon dioxide concentration at sites with significant WUE trends, and by vapor pressure deficit (VPD) at sites without significant trends (except cropland).

**Conclusions:**

Our findings revealed the commonalities and differences in the application of three WUE metrics in disparate ecosystems, and further highlighted the important effect of VPD on WUE change.

**Supplementary Information:**

The online version contains supplementary material available at 10.1186/s13021-023-00232-2.

## Background

Plants absorb atmospheric CO_2_ through photosynthesis at the cost of losing water through stomatal pores. Water use efficiency (WUE) is a critical metric to measure the tradeoff between carbon uptake and water use for terrestrial ecosystems in response to environmental changes [1]. At the ecosystem scale, WUE is typically defined as the ratio of gross primary productivity (GPP) to evapotranspiration (ET) [2]. WUE embodies the physiological and ecological characteristics of ecosystems, and provides important information for the interactions between carbon and water cycles. Elucidating the temporal dynamics of WUE in different ecosystems can help us better understand the response of ecosystem function to environmental change, improve our ability to predict ecosystem dynamics [3, 4], water availability [5, 6], and food production, and manage the biosphere to mitigate and adapt to climate change [7].

The temporal trends of WUE have been an important research topic under climate change. The results seem to vary among and even within ecosystem types. For example, WUE in forest ecosystems (1982–2012)[8], closed shrublands, and savannas was on the rise [9]; in contrast, WUE in open shrubs, woody savannas and grasslands showed an overall downward trend (2000–2013)[9]. Still, more studies suggested increasing WUE in forest (2000–2014)[10], grassland (2003–2006)[11], or cropland ecosystems and decreasing WUE for other grassland and cropland ecosystems (1981–2000 and 2001–2010)[12]. The discrepancies in the direction of WUE trends are likely caused by the specific ecosystems studied, the time periods investigated, different definitions of WUE [13], and differences in environmental responsiveness between the carbon and water cycles [14, 15]. Therefore, there is an urgent need to perform systematic comparative studies among different ecosystem types on the commonalties and differences of WUE change in response to changes in atmospheric and climate changes, using as many sites as possible.

Exploring the impact of atmospheric and climate factors (including CO_2_ concentration and meteorological factors) on WUE is a prerequisite for understanding the impact of climate change on the carbon-water cycle. Numerous studies have examined the response of WUE to elevated atmospheric CO_2_ concentrations and other factors using different approaches and datasets such as flux tower measurements [16, 17], field experiments [18], tree ring isotope measurements [8, 19–21], and model simulations [22, 23]. However, existing studies have documented inconsistent responses of WUE in different ecosystems to the same factors. Several studies have suggested that the WUE of oak forests in central Missouri, USA [24] and tropical rainforests in Xishuangbanna, Yunnan, China [25] are both affected by VPD while the WUE of the North China poplar plantations [26] and warm-temperate mixed forests [27] are both affected by SWC. A more recent study based on eddy covariance measurements at 44 sites worldwide showed that biotic and abiotic factors have differential effects on WUE in different ecosystem types, with VPD and canopy conductance playing important roles in controlling ecosystem WUE response to drought [28]. Nevertheless, there has been little discussion on how trends in atmospheric CO_2_ and meteorological variables based on eddy-covariance flux tower observations affect the temporal dynamics of WUE.

One of the challenges in comparing different WUE studies is their use of different WUE metrics. At least, three major metrics of WUE have been evolved over time and frequently used to measure the carbon-water tradeoff at the ecosystem scale [23, 29, 30]. The first is the so-called ecosystem water use efficiency (EWUE), defined at the ecosystem level as the ratio between GPP and ET [11, 31, 32]. Considering the intense impact of VPD on the carbon-water coupling [27, 33], Beer, Ciais [29] proposed the inherent water use efficiency (IWUE), defined as $IWUE=GPP\times \frac{VPD}{ET}$. IWUE depends on the ratio of intercellular (c_i_) to atmospheric (c_a_) concentrations of CO_2_, and is assumed to be relatively constant under given environmental conditions [34]. However, some studies have shown that IWUE varies with *c*_*i*_/*c*_*a*_ as VPD changes at the daily and seasonal timescales [25]. Thus, Zhou, Yu [35] proposed the underlying water use efficiency ($uWUE=GPP\times \frac{{VPD}^{0.5}}{ET}$) by combining IWUE using an optimal relationship between *c*_*i*_/*c*_*a*_ and VPD, and the authors indicated that the empirical relationship between GPP*VPD^0.5^ and ET in uWUE was more stable and more physiologically relevant than other WUE formulations. Essentially, these three WUE metrics all connect surface biological and physical processes [36], and can monitor the adaptation of ecosystems to changing climatic conditions [14, 17, 37, 38]. However, the use of different WUE metrics may yield different temporal trend and driving force attribution results. For example, the annual increase rate of IWUE was as high as 2.3% during the period 1995–2010 according to eddy-covariance measurements [17] while an analysis based on MODIS time series from 2000 to 2013 suggested that the average WUE decreased at a slope of -0.0045 g C kg^−1^ H_2_O yr^−1^ [39]. To date, a systematic comparison of the application in different WUE indicators remains absent.

In this study, we used the long-term records of carbon and water fluxes, and meteorological factors provided in the FLUXNET2015 dataset to analyze the trends of the three WUE metrics (i.e., EWUE, IWUE, uWUE) in forests (DBFs, ENFs), grasslands (GRAs) and croplands (CROs) and their responses to CO_2_ and climate perturbations. The specific objectives of our study were to: (1) simultaneously analyze the change trends of the three WUE metrics in three different types of ecosystems; (2) investigate the driving forces of WUE change trends in different ecosystems; and (3) explore the applicability of different WUE metrics in different ecosystems.

## Methods

### Study sites and data preparation

We used the daily carbon and water fluxes and meteorological data from the global flux network FLUXNET, including latent heat flux (LE), gross primary productivity (GPP), atmospheric CO_2_ concentration (Ca), air temperature (TA), vapor pressure deficit (VPD), shortwave radiation (SR) and soil water content (SWC). Data from 212 sites across the globe were retrieved from the FLUXNET2015 dataset (http://fluxnet.fluxdata.org/data/fluxnet2015-dataset/). We screened the data and sites according to the following criteria in combination with the corresponding QC patch files (https://fluxnet.org/data/fluxnet2015-dataset/known-issues/): (1) Eliminate invalid values of -9999 and retain high-quality data with QC > 75% [40], thereby only using reliable data; (2) Set the minimum values for GPP, ET, and VPD as follows: GPP > 0.1 g C d^− 1^ m^− 2^, ET > 0.05 mm d^− 1^, VPD > 0.001 kPa [41], in order to reduce the impact of random measurement errors on the carbon-water ratio when the observed fluxes were low; (3) Exclude days with precipitation events (P > 0.2 mm d^− 1^) and the day after rainfall to avoid the problems of rain interception and sensor saturation under high relative humidity [42]; (4) Select those sites with an observed length of more than 5 years [16]; (5) Exclude sites with energy balance closure ratio (Ra) values less than 0.60 or greater than 1.30 [43] to avoid energy imbalance issues at the sites used, i.e., the sum of the observed latent heat flux and sensible heat flux is different from the available energy, since all sites evaluated turbulent fluxes by eddy covariance. The Ra was calculated using the following formula (all units are in W m^− 2^):

1 $$Ra=\frac{LE+H}{Rn-G}$$

where H, Rn, and G are the sensible heat flux, surface net radiation and soil heat flux of the sites, respectively.

**Fig. 1:**
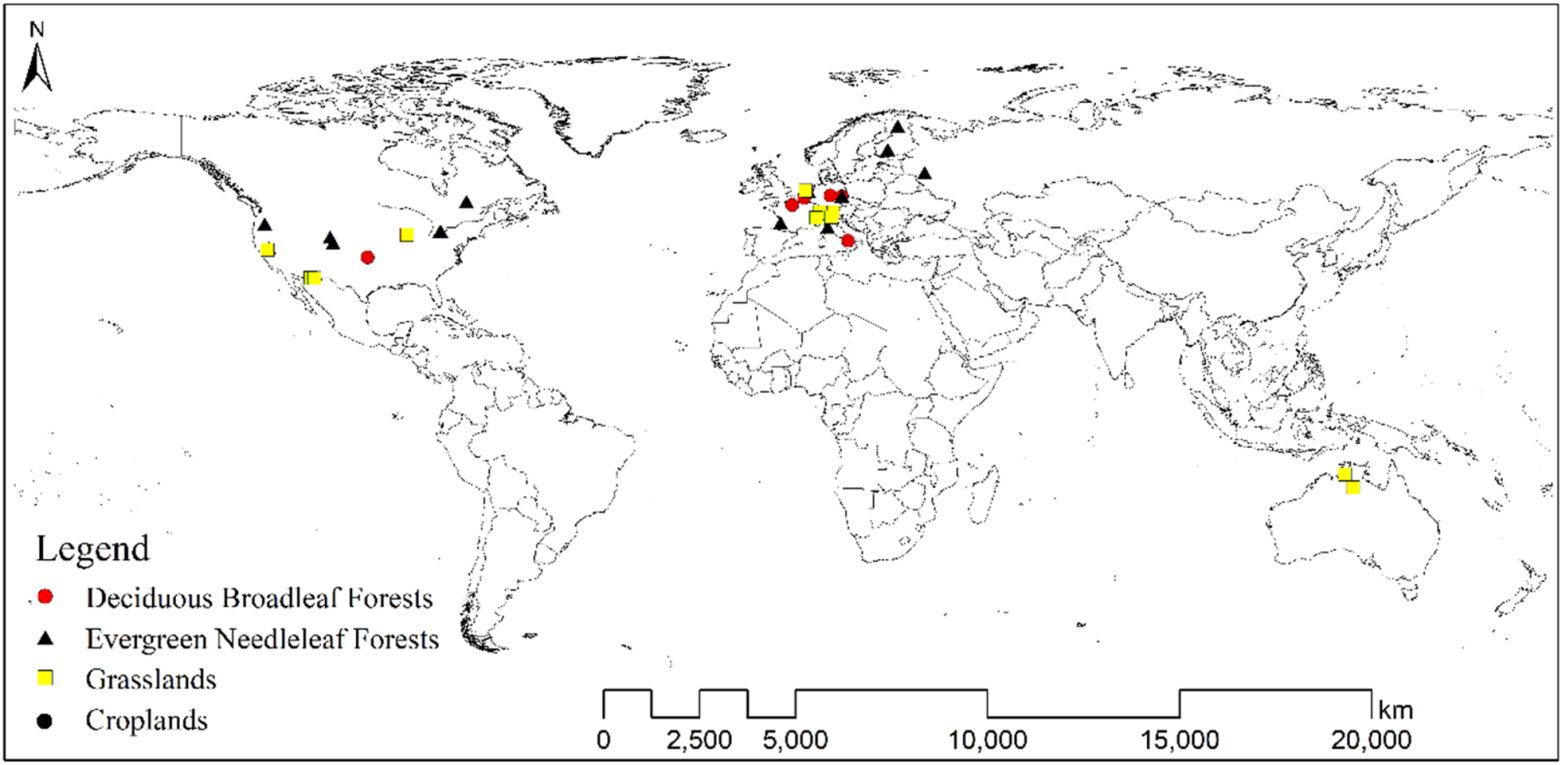
Distribution of the 42 FLUXNET sites used in this study. These sites fall into the following biomes: Deciduous Broadleaf Forests (*DBFs*), Evergreen Needleleaf Forests (*ENFs*), Grasslands (*GRAs*), and Croplands (*CROs*).

In this study, the biomes with fewer than 5 sites were eliminated, and finally the following four biomes with a total of 42 sites were retained for analysis: DBFs (7 sites), ENFs (18 sites), GRAs (11 sites), and CROs (6 sites) (Fig. 1 and Additional file 1: Table S1).

### Calculations of EWUE, IWUE and uWUE

We first calculated ET from LE. The form of calculating the ET using the eddy covariance method is as follows [44]:

2 $$ET=LE/\lambda \times 86,400$$

where ET is evapotranspiration (mm d^− 1^), LE is latent heat flux (w m^− 2^), and 86,400 is the time conversion coefficient (i.e., number of seconds in a day). The latent heat of vaporization $\lambda$ (MJ kg^− 1^) was calculated as follows Velpuri, Senay [45]:

3 $$\lambda =(2.51-0.00236t)\times {10}^{6}$$

where t is the temperature (^o^C),

In this study, EWUE is defined as:

4 $$EWUE=\frac{GPP}{ET}$$

where EWUE is ecosystem water use efficiency (g C kg^− 1^ H_2_O), and GPP is gross primary productivity (g C m^− 2^ d^− 1^).

IWUE (g C kPa kg^− 1^ H_2_O), defined in Beer, Ciais [29], was calculates as the EWUE multiplied by the mean daytime vapor pressure deficit (VPD, kPa):

5 $$IWUE=\frac{GPP\text{*}VPD}{ET}$$

The derivation of uWUE (g C kPa^0.5^ kg^− 1^ H_2_O) is based on IWUE and the optimization stomatal conductance model [46] to characterize the stable coupling relationship between GPP, ET and VPD [35]:

6 $$uWUE=\frac{GPP{\text{*}VPD}^{0.5}}{ET}$$

### Statistical analysis

The Mann-Kendall Trend Test is a common non-parametric method suitable for analyzing the time series data with continuous upward or downward trend (monotone trend). Since this method doesn’t require the raw data to obey a certain distribution and is insensitive to missing data or outliers, it has been widely used in hydrological and meteorological trend analysis [47, 48]. In this method, Z value was used to evaluate the statistical trend, with a positive Z value denoting an upward trend and a negative value indicating a downward trend. The *p* value obtained by the trend test denoted the significance level. The Sen’s slope estimator [8] was used to calculate the magnitude of trend for each variable at each site. Before trend detection, all variables were converted into relative annual changes (%, ratio of anomalies to multi-year mean values) to compare trends of different variables, following Keenan, Hollinger [17] and Wang, Chen [16]. We used the “trend” package in R for trend detection and slope estimation (https://cran.rproject.org/web/packages/trend/index.html). Then, one-way ANOVA with Fisher’s LSD test was used to assess the significance of the observed differences in trends across the biomes for the three WUE metrics.

We used the Bootstrap method based on previous research to calculate the average slope for each variable across biomes [49, 50]. Specifically, the slopes of all variables were resampled 10,000 times with replacement, then the mean values corresponding to each group of resampled data were calculated, and finally the 95% confidence intervals of the mean trends were calculated according to the obtained samples. The confidence intervals for the mean trend were estimated using the adjusted bootstrap percentile method provided by the “boot” package in R (https://cran.rproject.org/web/packages/boot/index.html).

The partial least square regression (PLSR) was used to investigate the responses of EWUE, IWUE and uWUE to atmospheric CO_2_ and meteorological factors (Ca, SR, TA, VPD and SWC). PLSR could effectively solve the problem of collinearity in independent variables by reducing the dimension of independent variables to obtain a smaller set of irrelevant components, and perform regression on these components [51] (Additional file 1: Table S10). All variables for each site were first converted to relative annual changes, and then the z-score of each variable for all sites was estimated so that regression coefficients of different independent variables could be compared. We used the “PLS” package in R to run the PLSR and to estimate the normalized coefficients and *p* values (https://cran.rproject.org/web/packages/pls/index.html). Lastly, to identify the one to two most important factors, the hierarchical partitioning analysis (HPA) method (R package “hier.part”: https://cran.rproject.org/web/packages/hier.part/index.html) was used [52].

## Results

### Temporal trends of WUE in forest, grassland and cropland ecosystems

**Fig. 2:**
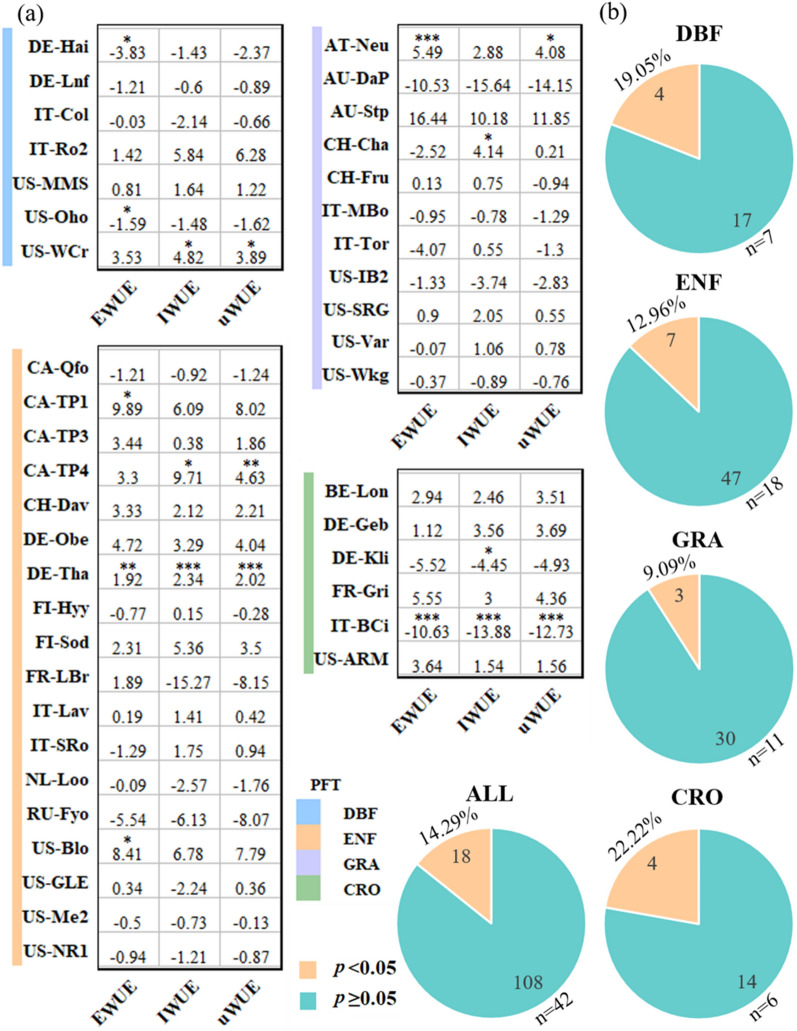
Trends (% yr^− 1^) in EWUE, IWUE and uWUE for all sites. **a** Trends and statistical significance of the three WUE metrics for all DBF, ENF, GRA, and CRO sites. The values in the squares represented the trends observed at each site for the three WUE metrics. Each * referred to a 0.05 for *p*, ** a 0.01 for *p*, *** a 0.001 for *p*. **b** Proportion of the number of significant (*p* < 0.05) and non-significant (*p* ≥ 0.05) trends for the three WUE metrics for each biome and all sites. n represents the number of sites

The WUE at most sites showed insignificant trends (Fig. 2 and Additional file 1: Table S2). Out of the 21 WUE trends calculated using all the three metrics at the seven DBF sites, only 4 of them or 19% were significant (2 positive trends and 2 negative trends) (*p* < 0.05). Only 7 out of 54 or 13% of the trends at the 18 ENF sites demonstrated significant trends (all positive). A mere 9% of the calculated WUE values showed significant trends at the 11 GRA sites (all positive). The CRO sites had a relatively higher fraction of significant WUE trends (all negative) than other biomes but still just at 22.2%. Pooling all WUE trends together across all sites from the four biomes, only about 14.3% of the WUE trends were significant (Fig. 2b).

### Consistency of the three WUE metrics in trends detection

**Fig. 3:**
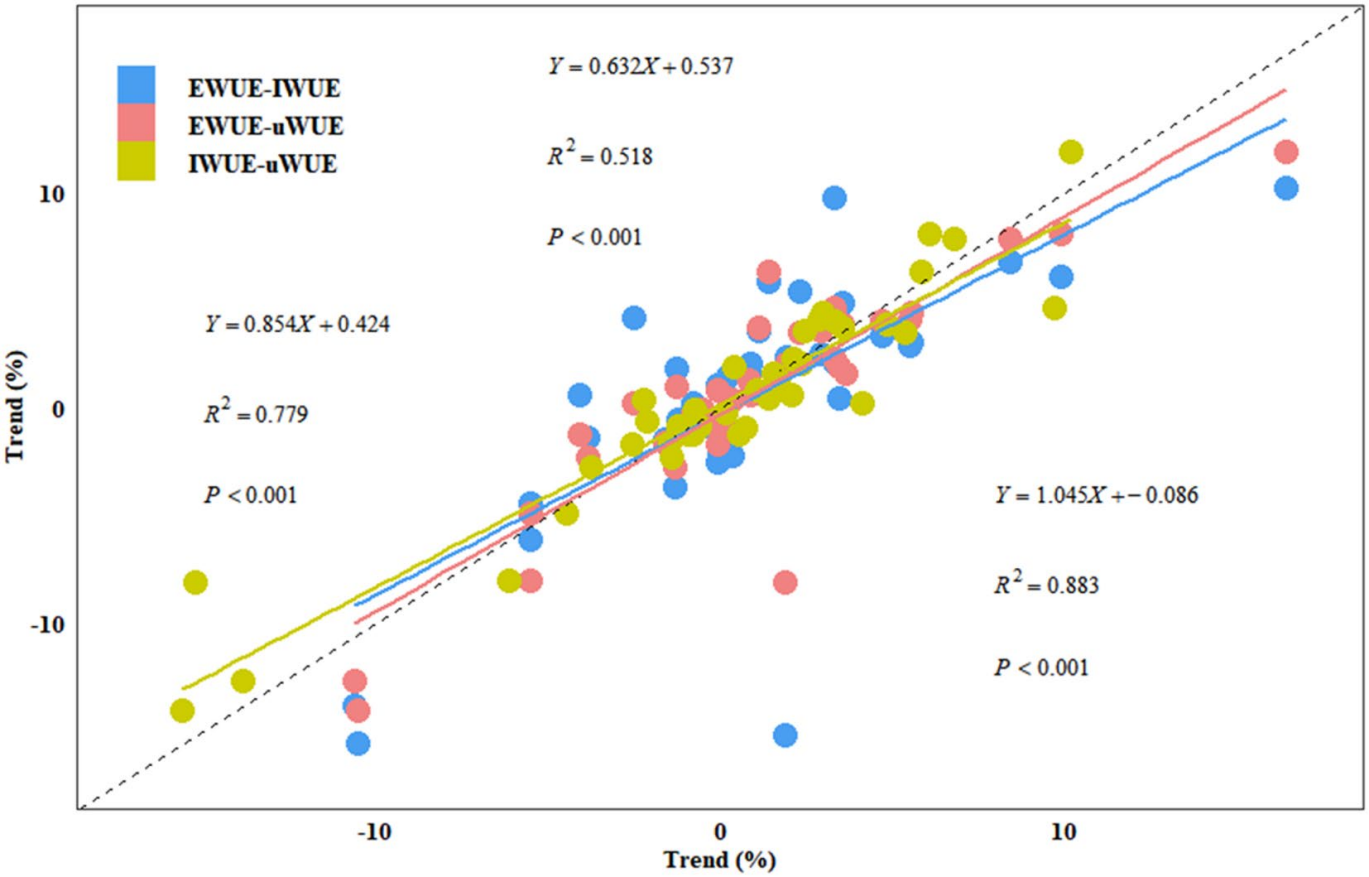
Comparison of trends (% yr^− 1^) obtained for the three metrics of WUE at different sites. Each scatter represents a trend obtained by pairs of mutually different WUE metrics. The dashed line shows the 1:1 line

The three WUE metrics showed high consistency in detecting no temporal trends across most of the sites, but demonstrated diverse performances in detecting significant trends (Fig. 2 and Additional file 1: Table S2). The significant trends detected with three WUE metrics were manifested in both DE-THa (ENF, EWUE: 1.92%; IWUE: 2.34%; uWUE: 2.02%) and IT-BCi (CRO, EWUE: -10.63%; IWUE: -13.88%; uWUE: -12.73%), while only 1–2 WUE metrics were effective when detecting significant trends for the residual 40 sites. The number of sites where one or more of the three WUE metrics detected a significant trend was actually quite large as well, accounting for about one-fourth (11/42) of the total number of sites. And, most of the sites with significant trends observed by one or two indicators were also similar to the non-significant results (*p*-value close to 0.1) of other WUE metrics in the corresponding sites. In addition, a modestly strong correlation was observed among EWUE-IWUE (R^2^ = 0.518, *p* < 0.001), EWUE-uWUE (R^2^ = 0.779, *p* < 0.001) and IWUE-uWUE (R^2^ = 0.883, *p* < 0.001) when monitoring trends at the ecosystem level (Fig. 3), but no WUE metric seemed systematically better than others in terms of performance (Fig. 2 and Additional file 1: Table S2). EWUE detected significantly at 7 (16.7%) of the 42 sites, and another two WUE metrics exhibited even poorer performance, with only 6 (IWUE: 14.3%) and 5 (uWUE: 11.9%) sites detected for significant trends, respectively. In parallel none of the three WUE metrics could capture a significant trend, this was represented across 31 (73.8%) sites (Fig. 2a).

**Fig. 4:**
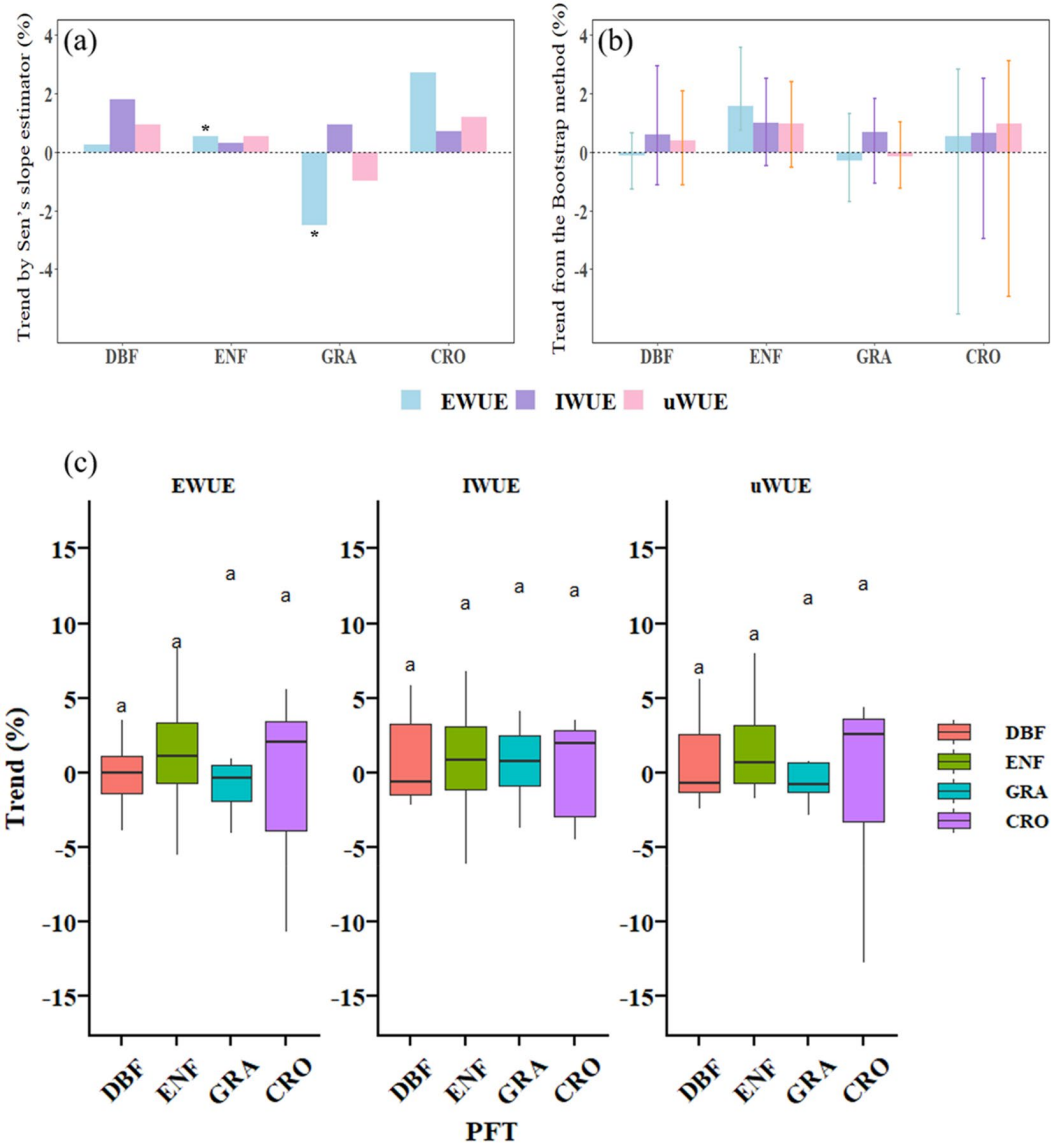
**a**, **b** Comparison of biome WUE changing trend (% yr^− 1^) from the Mann-Kendall + Sen Slope time series analysis method (annual slope [statistically significant values are indicated by star]; left panel) and the Bootstrap method (mean slope [the error bars represent the 95% confidence intervals of the mean trend]; right panel); **c** Comparison of the slope of EWUE, IWUE and uWUE at different sites (belonging to the same biome). The same lowercase letters indicated no statistically significant difference (LSD comparison)

Performance discrepancy could also be seen at the biome level. The observed EWUE based on the Sen’s slope estimator analysis method was significantly increased (slope = 0.53%, *p* < 0.05, 95% CI: 0.01–1.24) in ENF and significantly decreased (slope=-2.51%, *p* < 0.05, 95% CI: -4.81~-0.01) in GRA (Fig. 4a and Additional file 1: Table S3), while the WUE trends detected in the other two biomes (DBF and CRO) were non-statistically significant (Fig. 4a). Comparatively, the results obtained using the Bootstrap method indicated that only EWUE had a pronounced increasing trend (mean trend: 1.57%, 95% CI: 0.72 ~ 3.58) in ENF (Fig. 4b and Additional file 1: Table S4), and the remaining three biomes (DBF, GRA, and CRO) didn’t show a significant WUE trend, even at the 90% level was still nonsignificant (Additional file 1: Table S5). In the meantime, of the two accepted statistical analysis methods for assessing trends, none of the three WUE metrics simultaneously detected a significant trend in a biome. Moreover, according to the results of ANOVA for each vegetation type, there was no statistical difference (LSD’s test, *p* > 0.05) among the three WUE metrics in monitoring temporal trends among forest, GRA, and CRO ecosystems (Fig. 4c).

### Responses of EWUE, IWUE and uWUE to changes in atmospheric CO_2_ and meteorological factors

**Fig. 5:**
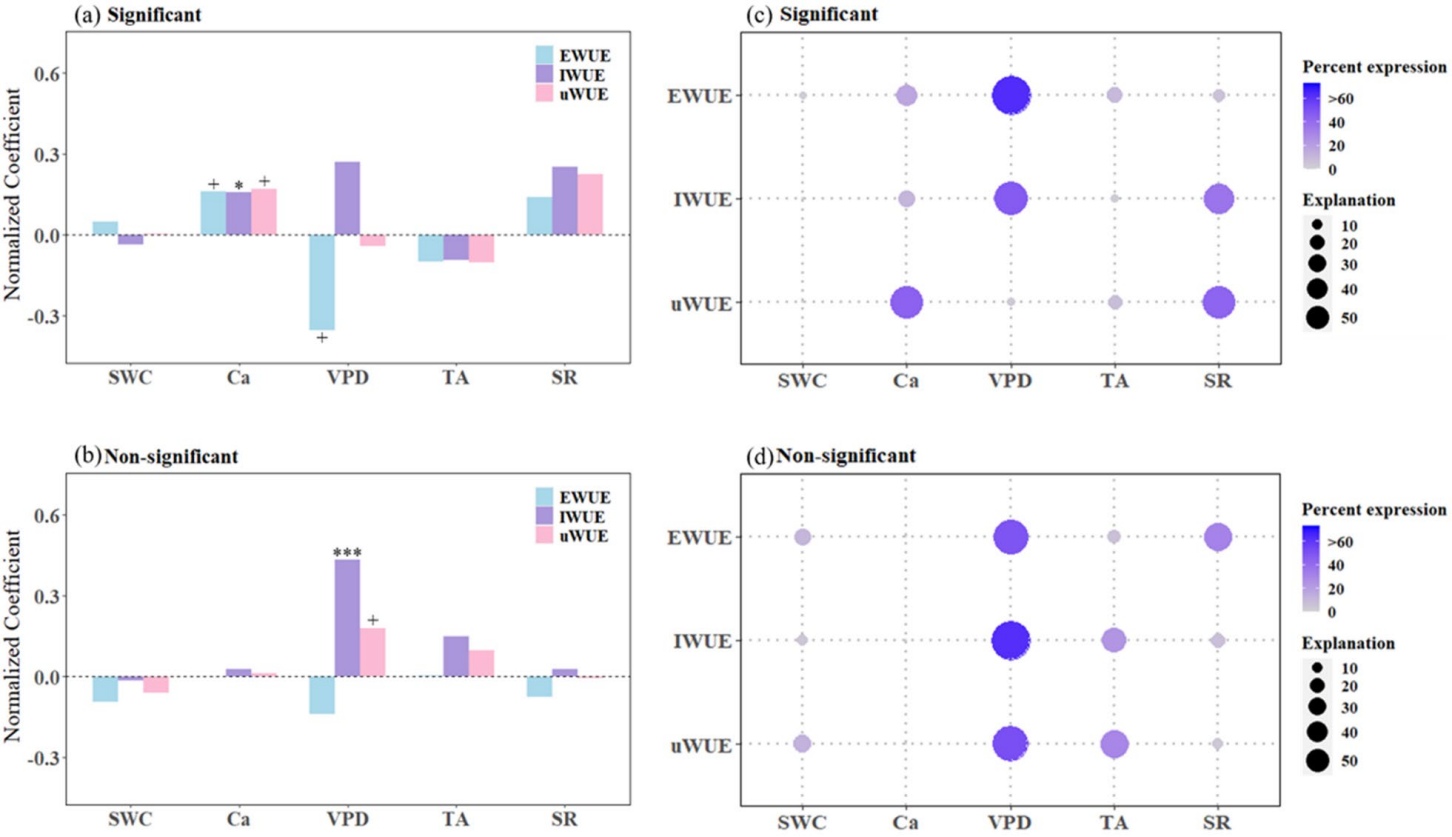
**a**, **b** Comparison of the response relationship between the trends of EWUE, IWUE and uWUE and trends of climate factors at the significant vs. non-significant sites. The marks above or below the bars indicate the significance of the regression: + indicates *p* < 0.1, * indicates *p* < 0.05, ** indicates *p* < 0.01, and *** indicates *p* < 0.001; **c**, **d** The relative importance of atmospheric CO_2_ concentration (Ca) and meteorological factors in influencing WUE at the significant and non-significant trend sites. The size of the circles represents the percentage of the variance that is explained by the independent variable, and the intensity of the color represents its importance

The factors affecting WUE change were inconsistent between significant and non-significant trend sites, but the most important factors for WUE were consistent (Fig. 5 and Additional file 1: Table S6). WUE at 11 sites with significant trends was mainly driven by Ca and VPD, while WUE trends at non-significant sites (31) responded significantly only to VPD. The HPA results indicated that VPD played the most important role in WUE changes at all sites (sig and non-sig), with the contribution rate reaching more than 60%.

The phenomenon that the three indicators were markedly associated with one independent variable at the same time existed in the sites with significant WUE trend, but the situation was just the opposite for the sites with a non-significant trend (Fig. 5a, b). At the sites with significant trend, EWUE (*p* < 0.1), IWUE (*p* < 0.05), uWUE (*p* < 0.1) showed strong positive correlations with Ca, with normalized regression coefficient of 0.161, 0.159, 0.17, individually. For non-significant trend sites, only IWUE (*p* < 0.001) and uWUE (*p* < 0.1) had significant positive effects on VPD. Unlike there, none of the independent variables at the significantly trending sites could have the highest importance for the three WUE metrics simultaneously (Fig. 5c and Additional file 1: Table S6). In specific, VPD was the most important factor for EWUE (63.84%) and IWUE (47.58%), while two factors were the most important for uWUE, Ca (44.63%) and SR (43.55%), because their interpretation rates are relatively close. And in non-significant trend sites, VPD had the highest relative importance among the three WUE metrics, which were 49.89% (EWUE), 63.32% (IWUE) and 52.06% (uWUE), respectively (Fig. 5d and Additional file 1: Table S8).

**Fig. 6:**
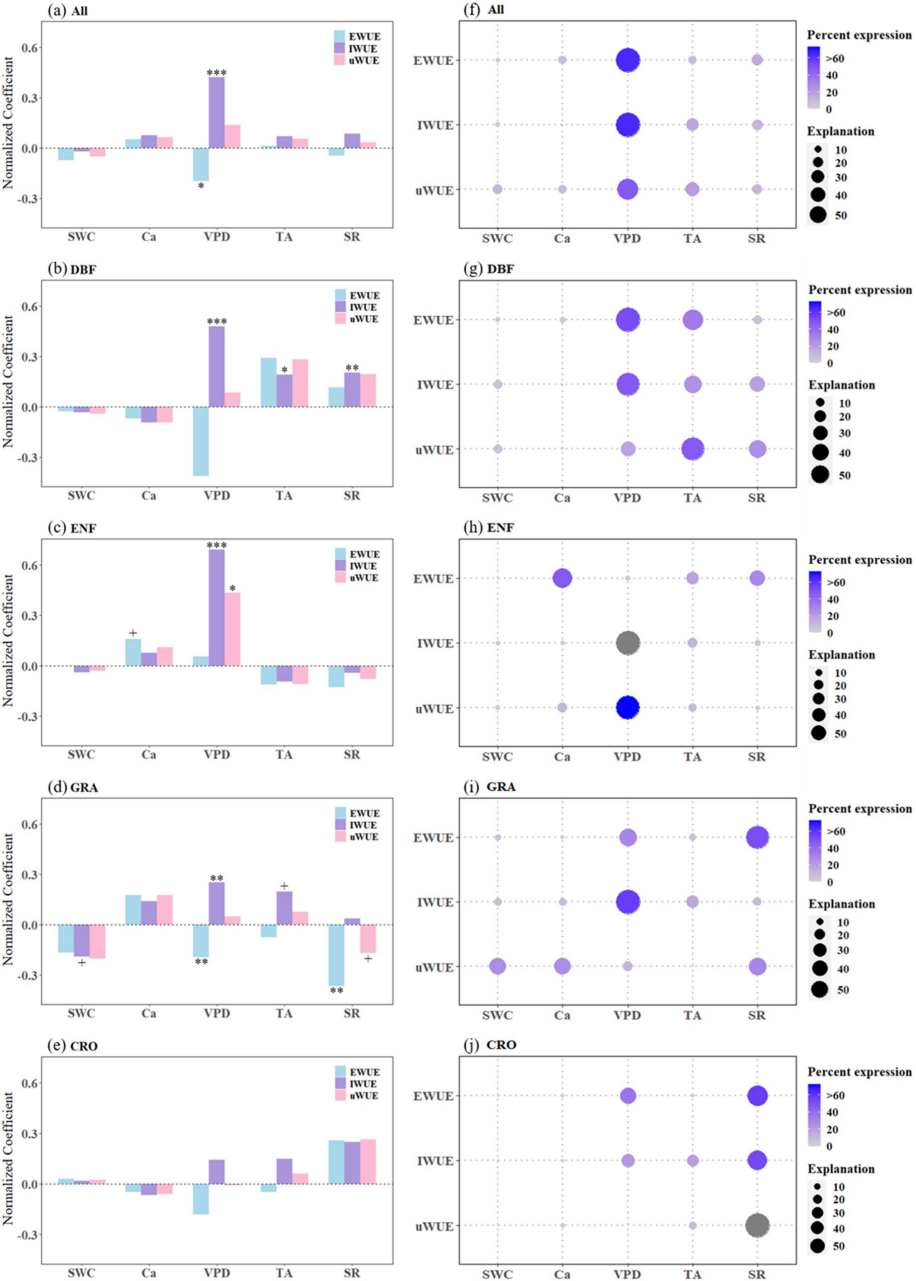
Normalized coefficients from the partial least squares regression between annual changes in EWUE, IWUE, uWUE and annual changes in Ca and meteorological variables for **a** all vegetation types (All), **b** DBFs, **c** ENFs, **d** GRAs and **e** CROs. The marks above or below the bars indicate the significance of the regression: + indicates *p* < 0.1, * indicates *p* < 0.05, ** indicates *p* < 0.01, and *** indicates *p* < 0.001. And the relative importance of Ca and meteorological variables in controlling EWUE, IWUE and uWUE in **f** all vegetation types (All), **g** DBFs, **h** ENFs, **i** GRAs and **j** CROs. The size of the circles represents the percentage of the variance that the dependent variable is explained by each independent variable, and the intensity of the color represents its importance

The effects of atmospheric CO_2_ and meteorological factors on WUE trends also varied across all vegetation types (All) and biomes. As a whole, WUE was strongly associated with VPD in All (Fig. 6a) and most biomes (except cropland). WUE was also significantly correlated with TA, SR in DBF and GRA (Fig. 6b, d), Ca in ENF (Fig. 6c) and SWC in GRA (Fig. 6d). Conversely, atmospheric CO_2_ and meteorological factors had no significant effect on the change of cropland WUE (Fig. 6e). Aside from this, none of the three WUE metrics had a pronounced response to one or some independent variables simultaneously. Only IWUE in All (*p* < 0.001), DBF (*p* < 0.001), ENF (*p* < 0.001) and GRA (*p* < 0.01) displayed a substantial positive correlation effect on VPD (Additional file 1: Table S7).

We further explored the relative importance of atmospheric CO_2_ and meteorological factors on the impact of the three WUE metrics in all vegetation types and different biomes using HPA, again with both commonalities and differences (Fig. 6f–j and Additional file 1: Table S9). Combining all vegetation types, the change of WUE was mainly affected by VPD (Fig. 6f), and the relative contribution rates of VPD to the changes of EWUE, IWUE and uWUE were 65.38%, 65.45% and 47.8%, respectively. At the biome scale, the WUE rate variation of CRO (EWUE: 58.19%, IWUE: 53.82% and uWUE: 86.92%) was mainly caused by SR (Fig. 6j), and the three WUE metrics of forest and GRA were dominated by different impact factors. To be specific, IWUE was mainly driven by VPD (DBF: 47.85%, ENF: 77.46%, GRA: 58.28%). EWUE (DBF: 52.64%) and uWUE (ENF: 72.83%) were mainly driven by VPD in forest, while SR was more important to EWUE (51.89%) and uWUE (30.6%) than other factors for GRA ecosystem. Meanwhile, the relative importance of Ca (47.52%) to the EWUE of the ENF and TA (47.66%) to the uWUE of the DBF were the highest, respectively.

## Discussion

### Insignificance of the WUE trends at most sites in forest, grassland, and cropland

Our results showed that the WUE trend was insignificant for most sites in forest, GRA and CRO. While observed carbon and water fluxes between the surface and the atmosphere using eddy-covariance could be used to imputed WUE trends [17, 53], these site datasets were always limited by uncertainty factors in measurement and method [54]. On the other hand, various WUE estimation methods might cause magnitude differences in temporal trends [55, 56], and yet, this difference was not shown in the significance test of the trend. Relevant studies showed that when various WUE metrics (e.g., Wei, IWUE, iWUE) were used to monitor the change trend of forest sites, most sites were not significant [16, 17, 54]. Furthermore, most of their WUE studies based on flux measurement were mostly locked in summer (June-August) or the peak period of gross ecosystem productivity [16, 57]. And the WUE obtained in this paper was based on the time axis of the growing season (April-September) of vegetation, which might pull down the annual mean value to some extent, but had no effect on testing whether the trend is significant. In total, the statistical data based on eddy-covariance technology verified that it was difficult to deduce reliable scientific conclusions from a single site or a few sites, i.e., most sites were not significant in trend monitoring. The simultaneous use of the three WUE metrics also exemplified that the test results didn’t deviate. It is believed that these data will be consolidated by keeping the trend study of different WUE definitions in the FLUXNET, as new flux tower datasets will be updated in the later iterations. On the other hand, the closure problem of the site energy balance derived from the uncertainty of latent heat observations would affect the robustness of the WUE slope estimation [58, 59], which might be the main reason why most trends were not significant.

It was also found in our study that while most sites showed insignificant trends, there were a few sites that exhibited highly diverse temporal trends in terms of magnitude and even direction of change. This discrepancy might be due to overestimation or underestimation derived from different WUE calculations and various observation periods at the sites [60]. For instance, the previous studies based on eddy covariance flux observations used diverse WUE metrics (iWUE = GEP/G_s_; Wei = W_e_D; IWUE = GEP*VPD/ET) at the same site to evaluate the trends presented [16, 17, 54], and the results were either higher or lower than the results of forest ecosystems in this study. Furthermore, studies have shown that despite wide local variability of WUE estimates, a convergence of WUE occurred across functional types in different geographic regions and climates [61], which was similar to what we observed in 26.2% of forest and grassland sites with significant trends. In particular, the majority of “+” trends (63.64% of significant trend sites) coincided (Fig. 2), illustrating long-term climatic conditions only partially controlled ecosystem WUE, and the increase of WUE might also mean the local plants were suffering from mild drought.

### Performance, variability and interchangeability of three metrics for assessing the WUE trends

It was demonstrated that three WUE metrics had the ability to capture temporal trends, but their performance varied across sites. The WUE equation in this paper was composed of GPP, GPP*VPD, GPP*VPD^0.5^ and ET individually, where EWUE usually exhibited relative consistency on the monthly to annual scale [29], and VPD changed rapidly on the daily to hourly scale, which had a significant impact on the carbon-water coupling process by regulating stomatal conductance [62]. With the introduction of VPD variable on the basis of GPP/ET, IWUE and uWUE performed more plausibly over shorter time-scales [35, 53]. Therefore, it could be seen that there would be performance differences when the three WUE metrics monitored the changing trend in the same time dimension. In essence, EWUE, IWUE and uWUE were linked by a common formula (GPP*VPD^k^/ET, where k = 0, 1 and 0.5, respectively), differing only in the value of the k-exponent [53]. Such as, oWUE (GPP*VPD^k*^/ET, k^*^ was the optimal exponential when the correlation coefficient between carbon and water was the highest) was not intrinsically better than uWUE in describing carbon-water coupling characteristics [35]. This meant that when different WUE metrics formed by the gradient change of k-value were compared and applied to specific ecosystems (sites), the processes shared similarities, which could be used to explain that the trends (significance) of the three WUE metrics were exceptionally similar in most sites. The finding validated the performance of the three WUE metrics when evaluating trends collectively, and underscored the importance of comparative studies, providing strong support for previous studies that focused on a single WUE metric [16, 17, 54]. Additionally, results from related studies that independently assessed trends at the same site using other different WUE indicators also demonstrated similarities and dissimilarities [17, 54]. This phenomenon was consistent with the results we displayed and was reflected in both grassland and cropland sites.

It was further found that the variation trends monitored by the three WUE metrics also differed among various biomes. We could see that WUE varied enormously among sites from this paper (Fig. 2), and previous studies have suggested that the combination of 20 sites could improve the robustness of the analysis results [54]. In view of the fact that the established FLUXNET2015 dataset wasn’t updated, all available sites with different monitoring lengths and spatial heterogeneity were bundled together to assess the overall trend of ecosystem-scale WUE, which greater highlighted the important role of statistical methods in estimating the trend of WUE [13]. In this paper, Sen’s slope estimator was used to determine the magnitude of the WUE trend in each biome [63], and Bootstrap statistical method was used to evaluate its trend for comparative study [64]. The application of the two methods in forest (ENF) was consistent with the results obtained by other WUE indicators, and the previous studies based on the FLUXNET2015 data likewise monitored a significant increase in iWUE (gross ecosystem productivity to canopy-scale water conductance) [16, 63]. Studies also existed that the temporal trend of WUE only in ENF, shrub and open shrubland was significant through the Bootstrap method, while there was no obvious change trend in other biomes [65], which further verified the difference in the results of diverse statistical methods applied in biomes. Concomitantly, it also proved that the true reliability of the trend might be directly affected by statistical methods, and its uncertainty needed more robust methods to verify and eliminate [54].

According to the above discussion, we didn’t identify a comparatively better WUE metric from this performance difference, implying that the three WUE metrics were interchangeable when used to detect trends. On the one hand, although each WUE metric could capture the significant trend of a few sites, the performance of the three was equivalent (Fig. 2), only monitoring the temporal trend that accounted for less than 20% of all sites. Simultaneously, the pairwise relationship between the three WUE metrics in different vegetation types sites were also strongly and positively correlated (Additional file 1: Fig. S1), indicating the possibility of substitution among different WUE metrics. In other terms, there was little difference in trend estimation using various WUE metrics at the same site. The performance superiority of the WUE metric might only be revealed when changes in WUE correlated with plant physiological behavior [55]. On the other hand, there were no significant differences in the trends monitored by the three WUE metrics in forest, GRA and CRO ecosystems (Fig. 4c), and this phenomenon has also been verified in the application of other diverse WUE metrics [13]. As suggested by Ponce Campos, Moran [66], different vegetation types might have intrinsic convergence in water use. Therefore, no matter which WUE equation was used to compare the trend differences among vegetation, there might be consistent results.

### Similarities and differences between in-situ WUE responses to climate change

It was found that the factors driving changes in WUE differed among sites (sig vs. non-sig), where EWUE, IWUE and uWUE of the significant trend sites were all significantly positively correlated with Ca. A previous study of forest ecosystems by Wang, Chen [16] found that decreased canopy conductance due to increased VPD in DBFs was the main contributor to increased WUE, while the increase of carbon uptake and limited stomatal constraint stimulated WUE growth in ENFs. Thus, WUE of different vegetation types had diverse sensitivity to various factors, which might be related to rooting depth, physiological structure, living conditions and drought resistance [28]. This further explained why the response factors differed between significant and insignificant trend sites. For sites with significant WUE trends, by themselves, might be more responsive to environmental changes [67]. Among the sites with significant trend in this paper, forest sites accounted for 63.6% (7/11), which was basically consistent with the findings of a study by Frank, Poulter [19] that forest WUE was positively correlated with the increase of Ca. In parallel, the same response of the three WUE metrics to Ca in the sites with significant trends explained to some extent the convergence of the three indicators when monitoring significant trends separately, and the consistent responses might be the product of the convergence results.

Our study also found similarities and differences in the responses of the three WUE metrics to Ca and meteorological factors between All and Biomes. WUE was mainly driven by VPD (except CRO) in the response analysis. This conclusion was supported by previous findings that revealed the increasing importance of VPD in ecosystem water and carbon fluxes [18, 68–70] and even suggested that vegetation change was dominated by moisture availability rather than Ca [16, 71]. This was also confirmed in the HPA results that VPD was the factor with the highest explanation rate of WUE (EWUE, IWUE, uWUE) in the Sig-Nonsig trend sites and All-Biomes, while the secondary factors were not the same (except CRO). Therefore, VPD is most likely to directly dominate the spatiotemporal pattern of WUE among all abiotic factors.

Beyond that the variation of WUE in CRO ecosystems was mainly caused by SR, but had no significant effect. Some studies have shown that non-climatic factors had a greater impact on the change of cropland WUE due to the disturbance of human factors [72], which might be the fundamental reason that no significant correlation between any natural factors and cropland ecosystem was observed in this paper. Hence, for CRO ecosystem, besides natural environmental factors, the impact of human activities (e.g., fertilization, irrigation, grazing) on WUE should also be considered.

### Potential limitations

Currently, the number of global flux tower sites is still limited, and the observation length is mostly short. Our study found a trumpet-shape between site monitoring length and change trend. The slope of sites with a length of over 12 years tended to be flattened, indicating that older sites were more stable in estimation, while sites below 12 years fluctuated greatly in rate variation, which might cause some deviations in trend assessment (Additional file 1: Fig. S2). Beside that the sites are unevenly distributed. Most of the sites were located in the northern hemisphere, and only two sites (Au-Dap, Au-stp) were scattered in Australia. When we tried to re-evaluate the trend after removing these two sites, the results showed that there was no significant change in the WUE trend, so it would not affect the overall conclusion drawn in this paper (Additional file 1: Fig. S3). Second, despite advances in EC systems and instrumentation, the energy balance closure problem of flux datasets still existed [73]. We screened the sites according to the corresponding standards, and the sites used were all within the applicable range of the energy closure rate to minimize the impact on the evaluation of WUE. Data collection and expansion should be strengthened in future study. Meanwhile, future research should also focus on the integration of ecological big data, promote the scientific exchange and cooperation of network observation data, remote sensing data, experimental data and other data, and help reveal the large-scale process mechanism and universal regularities of global prediction.

## Conclusions

In this paper, we used the Sen’s slope estimator method to investigate the temporal trends of three different WUE metrics in 42 sites, and compared the trends of four biomes with the Bootstrap method. In parallel, ANOVA was used to explore whether there were significant differences in the application of each indicator in various biomes. Finally, we used PLSR and HPA to explore the drivers of WUE changes and quantify their importance in the two dimensions of Sig-Nonsig trend sites and All-Biomes, respectively.

We found that the WUE of most sites in forest, grassland and cropland failed to capture temporal trends, and the application of the three WUE metrics between sites and biomes didn’t yield a relatively more applicable WUE indicator. In the meanwhile, there was no difference in the variation trends of the three WUE metrics among forest, grassland and cropland ecosystems. In addition to this, Ca had a significant positive effect on all three WUE metrics at the significant trend sites, whilst VPD played a dominant role (except CRO) in the remaining comparison groups (Non-sig, All and Biomes). The HPA analysis also demonstrated that VPD was the most important factor affecting the change of WUE (excluding CRO). For cropland ecosystem, SR had no significant effect in the response analysis, albeit it was the factor with the highest degree of explaining WUE trend. These results confirmed and supported some previous studies. With the possible intensification of future climate change, the simultaneous use of multiple WUE metrics to monitor temporal trends and their climate responses at different spatial scales can provide a scientific basis for us to better understand the relationship between vegetation change and the carbon-water cycle. And the importance of VPD in regulating vegetation WUE response to climate is emphasized.

## Supplementary Information

**Additional file 1. Figure S1.** Relationships between IWUE (**a**–**d**), uWUE (**e**–**h**) and EWUE, and uWUE (**i**–**l**) and IWUE in DBFs,ENFs, GRAs, CROs. **Figure S2.** Distribution of differences between WUE changes and the number of years at all sites (*p*<0.1). **Figure S3. **The linear change trend of ecosystem water use efficiency (EWUE; **a**), inherent water use efficiency (IWUE; **b**) and underlying water use efficiency (uWUE; **c**) in grasslands (GRAs). The gray dots and black lines represent the relative annual changes and regression lines of the sites, respectively. The slope is derived from each site by Sen’s slope estimator. The thick red line represents the average trend of all sites. The upper left illustration shows the frequency distribution of slopes for all sites. The vertical red dashed line represents the 95% confidence interval of the average slope. **Table S1.** Site information, including the site codes (site), country, forest functional types as defined by the International Geosphere Biosphere Program (IGBP), latitude, longitude, elevation, duration and EBR (energy balance closure ratio). At least 5 available site years for the response analysis. **Table S2. **Trends of EWUE, IWUE and uWUE for all DBF, ENF, GRA and CRO sites. **Table S3.** EWUE, IWUE and uWUE trends of DBF, ENF, GRA and CRO obtained by Sen's slope estimator method. **Table S4. **EWUE, IWUE and uWUE trends of DBF, ENF, GRA and CRO obtained by Bootstrap method. Cilow and Cihigh represent 95% confidence intervals for trends. **Table S5.** EWUE, IWUE and uWUE trends of DBF, ENF, GRA and CRO obtained by Bootstrap method. Cilow and Cihigh represent 90% confidence intervals for trends. **Table S6. **Normalized coefficients from PLSR between the annual anomalies of EWUE/IWUE/uWUE and meteorological variables for Significant and Non-significant sites. **Table S7.** Normalized coefficients from PLSR between theannual anomalies of EWUE/IWUE/uWUE and meteorological variables for ALLs, DBFs,ENFs, GRAs and CROs. **Table S8. **The relative importance of meteorological variables to dependent variables (EWUE, IWUE, uWUE) under different sites (Significant, Non-significant). **Table S9. **The relative importance of meteorological variables to dependent variables (EWUE, IWUE, uWUE) under different biomes (ALL, DBF, ENF, GRA, CRO). **Table S10.** Correlation coefficients between the independent variables for DBFs, ENFs, GRAs and CROs.

## Data Availability

All Eddy covariance data are available from http://fluxnet.fluxdata.org/data/fluxnet2015-dataset (last access: 26 April 2017).
