# Supplementary material for: Temporal dynamics of ecosystem, inherent, and underlying water use efficiencies of forests, grasslands, and croplands and their responses to climate change
Source: Carbon Balance Manag. 2023 Jul 14;18:13. doi: 10.1186/s13021-023-00232-2 (PMC10347772; doi:10.1186/s13021-023-00232-2)
Supplement: Supplementary file 1 — Additional file 1. Figure S1. Relationships between IWUE (a–d), uWUE (e–h) and EWUE, and uWUE (i–l) and IWUE in DBFs,ENFs, GRAs, CROs. Figure S2. Distribution of differences between WUE changes and the number of years at all sites (p<0.1). Figure S3. The linear change trend of ecosystem water use efficiency (EWUE; a), inherent water use efficiency (IWUE; b) and underlying water use efficiency (uWUE; c) in grasslands (GRAs). The gray dots and black lines represent the relative annual changes and regression lines of the sites, respectively. The slope is derived from each site by Sen’s slope estimator. The thick red line represents the average trend of all sites. The upper left illustration shows the frequency distribution of slopes for all sites. The vertical red dashed line represents the 95% confidence interval of the average slope. Table S1. Site information, including the site codes (site), country, forest functional types as defined by the International Geosphere Biosphere Program (IGBP), latitude, longitude, elevation, duration and EBR (energy balance closure ratio). At least 5 available site years for the response analysis. Table S2. Trends of EWUE, IWUE and uWUE for all DBF, ENF, GRA and CRO sites. Table S3. EWUE, IWUE and uWUE trends of DBF, ENF, GRA and CRO obtained by Sen's slope estimator method. Table S4. EWUE, IWUE and uWUE trends of DBF, ENF, GRA and CRO obtained by Bootstrap method. Cilow and Cihigh represent 95% confidence intervals for trends. Table S5. EWUE, IWUE and uWUE trends of DBF, ENF, GRA and CRO obtained by Bootstrap method. Cilow and Cihigh represent 90% confidence intervals for trends. Table S6. Normalized coefficients from PLSR between the annual anomalies of EWUE/IWUE/uWUE and meteorological variables for Significant and Non-significant sites. Table S7. Normalized coefficients from PLSR between theannual anomalies of EWUE/IWUE/uWUE and meteorological variables for ALLs, DBFs,ENFs, GRAs and CROs. Table S8. The relative importance of meteo [file 13021_2023_232_MOESM1_ESM.docx]

# Additional file 1

**Figure S1.** Relationships between IWUE (a, b, c, d), uWUE (e, f, g, h) and EWUE, and uWUE (i, j, k, l) and IWUE in DBFs, ENFs, GRAs, CROs.

**Figure S2.** Distribution of differences between WUE changes and the number of years at all sites (*p*<0.1).

**Figure S3.** The linear change trend of ecosystem water use efficiency (EWUE; a), inherent water use efficiency (IWUE; b) and underlying water use efficiency (uWUE; c) in grasslands (GRAs). The gray dots and black lines represent the relative annual changes and regression lines of the sites, respectively. The slope is derived from each site by Sen’s slope estimator. The thick red line represents the average trend of all sites. The upper left illustration shows the frequency distribution of slopes for all sites. The vertical red dashed line represents the 95% confidence interval of the average slope.

**Table S1.** Site information, including the site codes (site), country, forest functional types as defined by the International Geosphere Biosphere Program (IGBP), latitude, longitude, elevation, duration and EBR (energy balance closure ratio). At least 5 available site years for the response analysis.

**Table S2.** Trends of EWUE, IWUE and uWUE for all DBF, ENF, GRA and CRO sites.

**Table S3.** EWUE, IWUE and uWUE trends of DBF, ENF, GRA and CRO obtained by Sen's slope estimator method.

**Table S4.** EWUE, IWUE and uWUE trends of DBF, ENF, GRA and CRO obtained by Bootstrap method. Cilow and Cihigh represent 95% confidence intervals for trends.

**Table S5.** EWUE, IWUE and uWUE trends of DBF, ENF, GRA and CRO obtained by Bootstrap method. Cilow and Cihigh represent 90% confidence intervals for trends.

**Table S6.** Normalized coefficients from PLSR between the annual anomalies of EWUE/IWUE/uWUE and meteorological variables for Significant and Non-significant sites.

**Table S7.** Normalized coefficients from PLSR between the annual anomalies of EWUE/IWUE/uWUE and meteorological variables for ALLs, DBFs, ENFs, GRAs and CROs.

**Table S8.** The relative importance of meteorological variables to dependent variables (EWUE, IWUE, uWUE) under different sites (Significant, Non-significant).

**Table S9.** The relative importance of meteorological variables to dependent variables (EWUE, IWUE, uWUE) under different biomes (ALL, DBF, ENF, GRA, CRO).

**Table S10.** Correlation coefficients between the independent variables for DBFs, ENFs, GRAs and CROs.


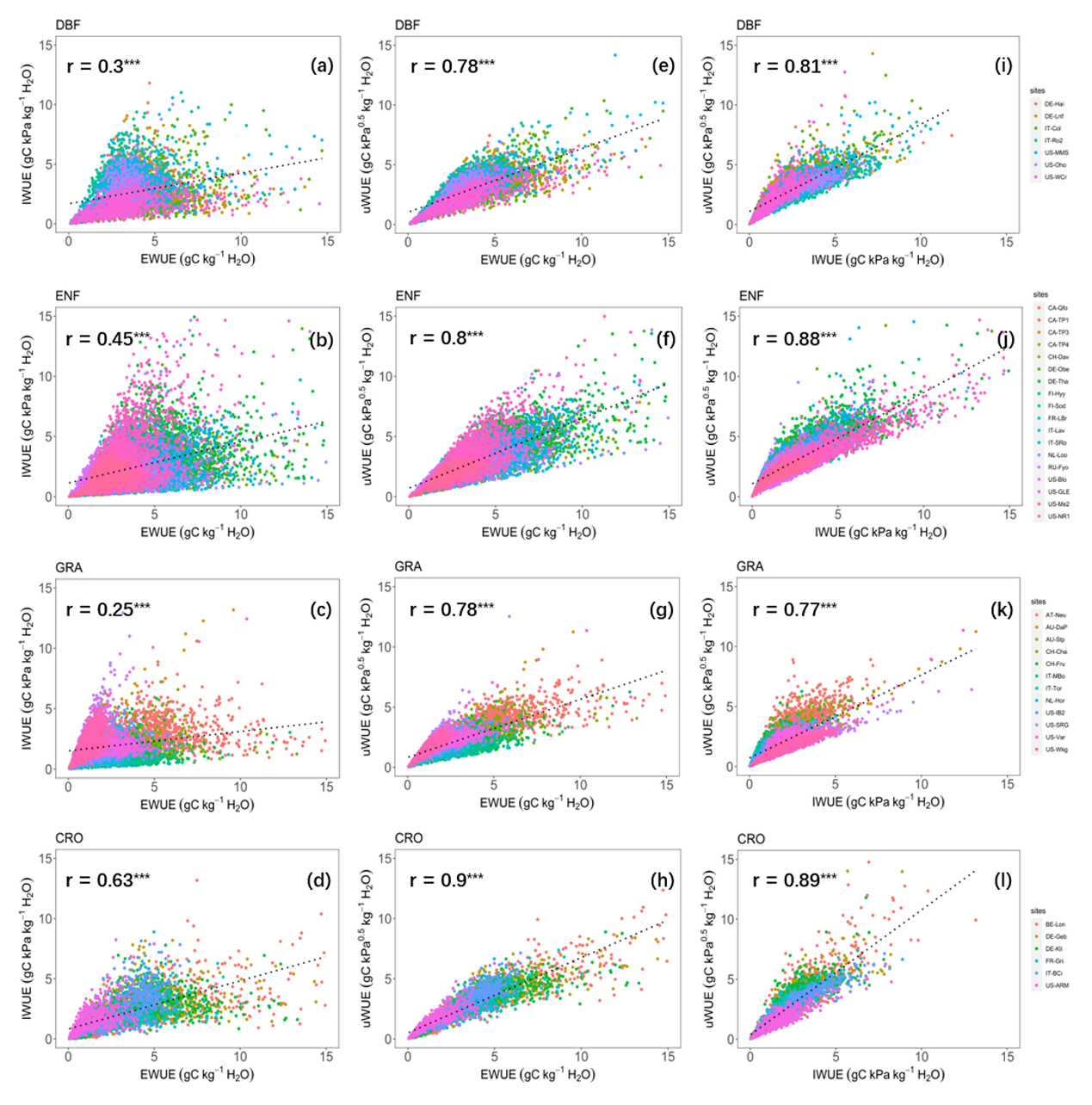


**Figure S1.** Relationships between IWUE (a, b, c, d), uWUE (e, f, g, h) and EWUE, and uWUE (i, j, k, l) and IWUE in DBFs, ENFs, GRAs, CROs. The dotted line is calculated using a linear regression fit method. Different colors represent different sites. r represents the total correlation coefficient between IWUE, uWUE and EWUE, and uWUE and IWUE in various vegetation types. “***” indicates *p* value less than 0.001.


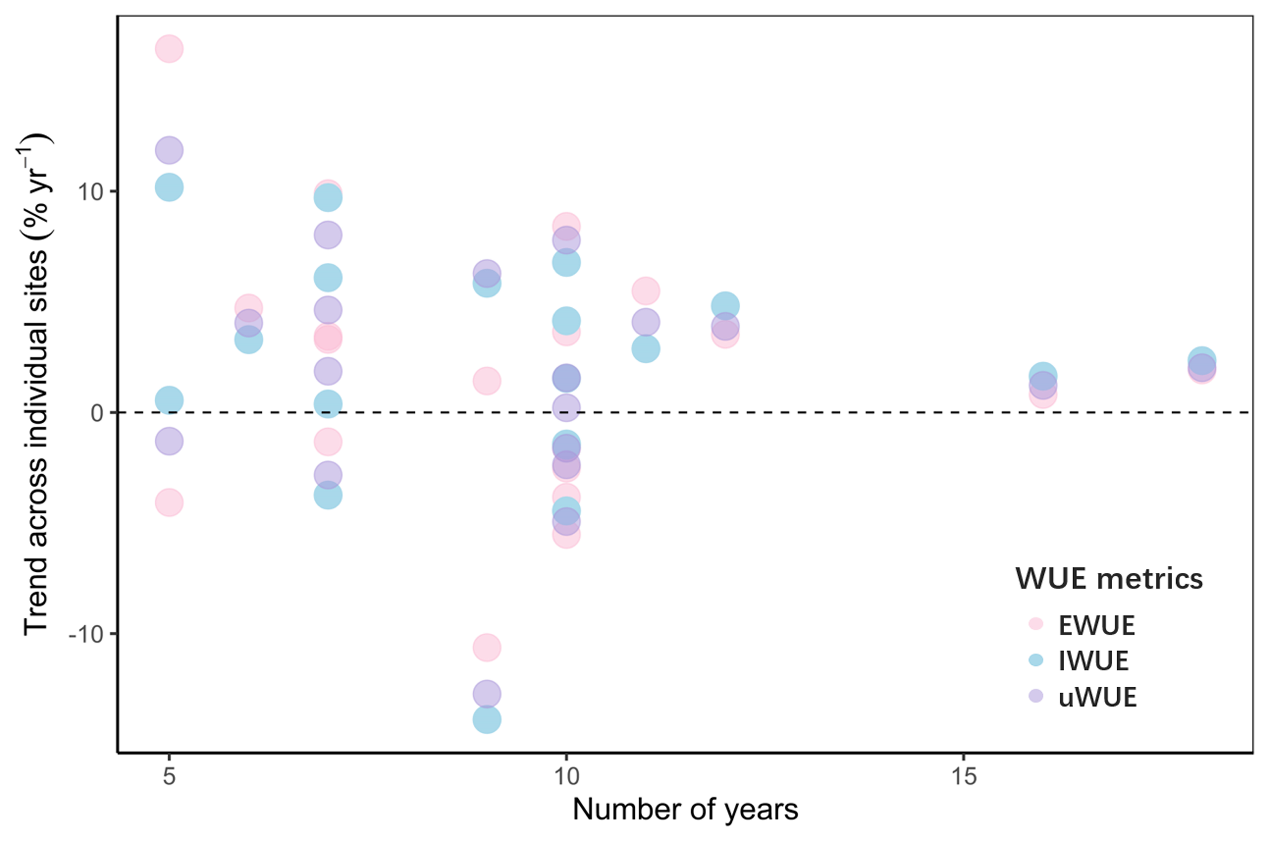


**Figure S2.** Distribution of differences between WUE changes and the number of years at all sites (*p*<0.1).


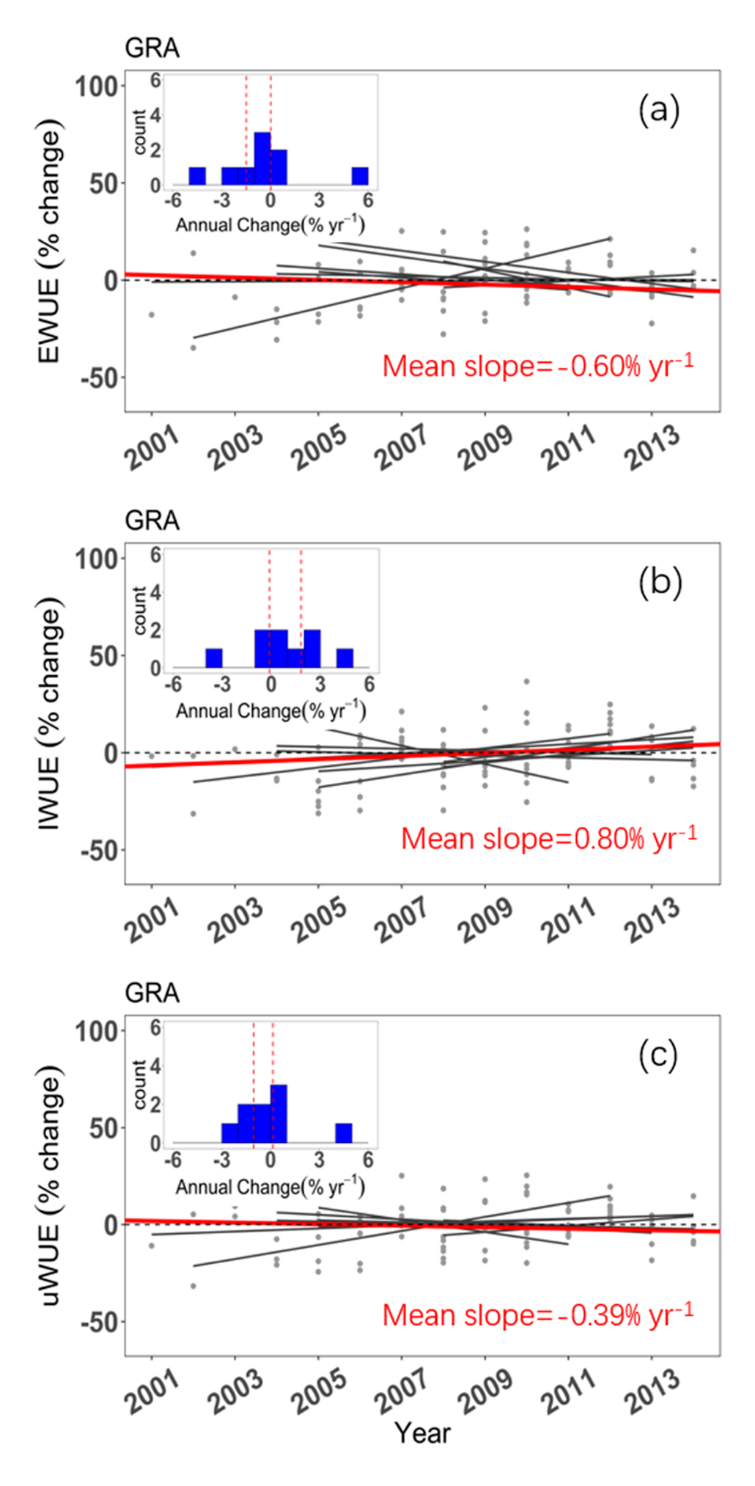


**Figure S3.** The linear change trend of ecosystem water use efficiency (EWUE; a), inherent water use efficiency (IWUE; b) and underlying water use efficiency (uWUE; c) in grasslands (GRAs). The gray dots and black lines represent the relative annual changes and regression lines of the sites, respectively. The slope is derived from each site by Sen’s slope estimator. The thick red line represents the average trend of all sites. The upper left illustration shows the frequency distribution of slopes for all sites. The vertical red dashed line represents the 95% confidence interval of the average slope.

**Table S1.** Site information, including the site codes (site), country, forest functional types as defined by the International Geosphere Biosphere Program (IGBP), latitude, longitude, duration and EBR (energy balance closure ratio). At least 5 available site years for the response analysis.

| Site | Country | IGBP | Latitude | Longitude | Duration | EBR | Reference |
| --- | --- | --- | --- | --- | --- | --- | --- |
|  |  |  | (°) | (°) |  |  |  |
| DE-Hai | Germany | DBF | 51.079 | 10.453 | 2000-2012 | 0.816 | (Knohl et al., 2003) |
| DE-Lnf | Germany | DBF | 51.328 | 10.368 | 2002-2012 | 0.846 | (Anthoni et al., 2004) |
| IT-Col | Italy | DBF | 41.849 | 13.588 | 1996-2014 | 0.654 | (R. VALENTINI et al., 1996) |
| IT-Ro2 | Italy | DBF | 42.390 | 11.921 | 2002-2012 | 0.666 | (Tedeschi et al., 2006) |
| US-MMS | USA | DBF | 39.323 | -86.413 | 1999-2014 | 0.788 | (Baldocchi et al., 2005) |
| US-Oho | USA | DBF | 41.555 | -83.844 | 2004-2013 | 0.777 | (Noormets et al., 2008) |
| US-WCr | USA | DBF | 45.806 | -90.080 | 1999-2014 | 0.762 | (Cook et al., 2004) |
| CA-Qfo | Canada | ENF | 49.693 | -74.342 | 2003-2010 | 0.734 | (Bergeron et al., 2007) |
| CA-TP1 | Canada | ENF | 42.661 | -80.560 | 2002-2014 | 0.807 | (Peichl et al., 2010) |
| CA-TP3 | Canada | ENF | 42.707 | -80.348 | 2002-2014 | 0.852 | (Peichl et al., 2010) |
| CA-TP4 | Canada | ENF | 42.710 | -80.357 | 2002-2014 | 0.694 | (Arain and Restrepo-Coupe, 2005) |
| CH-Dav | Switzerland | ENF | 46.815 | 9.856 | 1997-2014 | 0.624 | (Zielis et al., 2014) |
| DE-Obe | Germany | ENF | 50.784 | 13.720 | 2008-2014 | 0.798 | (Zimmermann et al., 2006) |
| DE-Tha | Germany | ENF | 50.964 | 13.567 | 1996-2014 | 0.852 | (GrüNwald and Bernhofer, 2017) |
| FI-Hyy | Finland | ENF | 61.848 | 24.295 | 1996-2014 | 0.833 | (Vesala et al., 2005) |
| FI-Sod | Finland | ENF | 67.362 | 26.638 | 2001-2014 | 1.070 | (Thum et al., 2007) |
| FR-LBr | France | ENF | 44.717 | -0.769 | 1996-2008 | 0.793 | (Paul Berbigier et al., 2001) |
| IT-Lav | Italy | ENF | 45.956 | 11.281 | 2003-2014 | 0.803 | (B. Marcolla et al., 2003) |
| IT-SRo | Italy | ENF | 43.728 | 10.284 | 1999-2012 | 1.084 | (Montagnani et al., 2009) |
| NL-Loo | Netherlands | ENF | 52.167 | 5.744 | 1996-2014 | 0.783 | (Stoy et al., 2014) |
| RU-Fyo | Russia | ENF | 56.462 | 32.922 | 1998-2014 | 0.887 | (J. Kurbatova et al., 2008) |
| US-Blo | USA | ENF | 38.895 | -120.633 | 1997-2007 | 0.729 | (R. Holzinger et al., 2006) |
| US-GLE | USA | ENF | 41.367 | -106.240 | 2004-2014 | 0.844 | (Frank et al., 2014) |
| US-Me2 | USA | ENF | 44.452 | -121.557 | 2002-2014 | 0.708 | (Vickers et al., 2009) |
| US-NR1 | USA | ENF | 40.033 | -105.546 | 1998-2014 | 0.823 | (R. K. MONSON et al., 2002) |
| AT-Neu | Austria | GRA | 47.117 | 11.318 | 2002-2012 | 0.776 | (Wohlfahrt et al., 2008) |
| AU-DaP | Australia | GRA | -14.063 | 131.318 | 2007-2013 | 0.902 | (Beringer et al., 2011) |
| AU-Stp | Australia | GRA | -17.151 | 133.350 | 2008-2014 | 0.977 | (Beringer et al., 2011) |
| CH-Cha | Switzerland | GRA | 47.210 | 8.410 | 2005-2014 | 0.976 | (Eugster and Zeeman, 2006) |
| CH-Fru | Switzerland | GRA | 47.116 | 8.538 | 2005-2014 | 0.909 | (Eugster and Zeeman, 2006) |
| IT-MBo | Italy | GRA | 46.015 | 11.046 | 2003-2013 | 0.941 | (Marcolla et al., 2011) |
| IT-Tor | Italy | GRA | 45.844 | 7.578 | 2008-2014 | 0.982 | (Galvagno et al., 2013) |
| US-IB2 | USA | GRA | 41.841 | -88.241 | 2004-2011 | 0.668 | (Matamala et al., 2008) |
| US-SRG | USA | GRA | 31.789 | -110.828 | 2008-2014 | 0.892 | (Scott et al., 2015) |
| US-Var | USA | GRA | 38.413 | -120.951 | 2000-2014 | 0.957 | (Ma et al., 2007) |
| US-Wkg | USA | GRA | 31.737 | -109.942 | 2004-2014 | 0.946 | (Scott, 2016) |
| BE-Lon | Belgium | CRO | 50.552 | 4.746 | 2004-2014 | 0.642 | (Moureaux et al., 2006) |
| DE-Geb | Germany | CRO | 51.100 | 10.914 | 2001-2014 | 0.838 | (Anthoni et al., 2004) |
| DE-Kli | Germany | CRO | 50.893 | 13.523 | 2004-2014 | 0.650 | (Ceschia et al., 2010) |
| FR-Gri | France | CRO | 48.844 | 1.952 | 2004-2014 | 0.935 | (Loubet et al., 2011) |
| IT-BCi | Italy | CRO | 40.524 | 14.957 | 2004-2014 | 0.985 | (Vitale et al., 2016) |
| US-ARM | USA | CRO | 36.606 | -97.489 | 2003-2012 | 0.600 | (Fischer et al., 2007) |

**References of sites**

Anthoni, P., Knohl, A., Rebmann, C., Freibauer, A., Mund, M., Ziegler, W., Kolle, O.,Schulze, E.D., 2004. Forest and agricultural land‐use‐dependent CO2 exchange in Thuringia, Germany. Global Change Biol., 10(12): 2005-2019.

Arain, M.A.,Restrepo-Coupe, N., 2005. Net ecosystem production in a temperate pine plantation in southeastern Canada. Agricultural and Forest Meteorology, 128(3-4): 223-241.

B. Marcolla, A. Pitacco,Cescatti, A., 2003. Canopy architecture and turbulence structure in a coniferous forest. Boundary-Layer Meteorology volume, 108(1): 39-59.

Baldocchi, D.D., Black, T.A., Curtis, P.S., Falge, E., Fuentes, J.D., Granier, A., Gu, L., Knohl, A., Pilegaard, K., Schmid, H.P., Valentini, R., Wilson, K., Wofsy, S., Xu, L.,Yamamoto, S., 2005. Predicting the onset of net carbon uptake by deciduous forests with soil temperature and climate data: a synthesis of FLUXNET data. Int J Biometeorol, 49(6): 377-87.

Bergeron, O., Margolis, H.A., Black, T.A., Coursolle, C., Dunn, A.L., Barr, A.G.,Wofsy, S.C., 2007. Comparison of carbon dioxide fluxes over three boreal black spruce forests in Canada. Global Change Biol., 13(1): 89-107.

Beringer, J., Hutley, L.B., Hacker, J.M., Neininger, B.,Paw U, K.T., 2011. Patterns and processes of carbon, water and energy cycles across northern Australian landscapes: From point to region. Agricultural and Forest Meteorology, 151(11): 1409-1416.

Ceschia, E., Béziat, P., Dejoux, J.F., Aubinet, M., Bernhofer, C., Bodson, B., Buchmann, N., Carrara, A., Cellier, P., Di Tommasi, P., Elbers, J.A., Eugster, W., Grünwald, T., Jacobs, C.M.J., Jans, W.W.P., Jones, M., Kutsch, W., Lanigan, G., Magliulo, E., Marloie, O., Moors, E.J., Moureaux, C., Olioso, A., Osborne, B., Sanz, M.J., Saunders, M., Smith, P., Soegaard, H.,Wattenbach, M., 2010. Management effects on net ecosystem carbon and GHG budgets at European crop sites. Agric., Ecosyst. Environ., 139(3): 363-383.

Cook, B.D., Davis, K.J., Wang, W., Desai, A., Berger, B.W., Teclaw, R.M., Martin, J.G., Bolstad, P.V., Bakwin, P.S., Yi, C.,Heilman, W., 2004. Carbon exchange and venting anomalies in an upland deciduous forest in northern Wisconsin, USA. Agricultural and Forest Meteorology, 126(3-4): 271-295.

Eugster, W.,Zeeman, M.J., 2006. Micrometeorological techniques to measure ecosystem-scale greenhouse gas fluxes for model validation and improvement. Int Congr Ser, 1293: 66-75.

Fischer, M.L., Billesbach, D.P., Berry, J.A., Riley, W.J.,Torn, M.S., 2007. Spatiotemporal Variations in Growing Season Exchanges of CO2, H2O, and Sensible Heat in Agricultural Fields of the Southern Great Plains. Earth Interactions, 11(17): 1-21.

Frank, J.M., Massman, W.J., Ewers, B.E., Huckaby, L.S.,Negrón, J.F., 2014. Ecosystem CO2/H2O fluxes are explained by hydraulically limited gas exchange during tree mortality from spruce bark beetles. Journal of Geophysical Research: Biogeosciences, 119(6): 1195-1215.

Galvagno, M., Wohlfahrt, G., Cremonese, E., Rossini, M., Colombo, R., Filippa, G., Julitta, T., Manca, G., Siniscalco, C., Morra di Cella, U.,Migliavacca, M., 2013. Phenology and carbon dioxide source/sink strength of a subalpine grassland in response to an exceptionally short snow season. Environmental Research Letters, 8(2).

GrüNwald, T.,Bernhofer, C., 2017. A decade of carbon, water and energy flux measurements of an old spruce forest at the Anchor Station Tharandt. Tellus B: Chemical and Physical Meteorology, 59(3): 387-396.

J. Kurbatova, C. Li, A. Varlagin, X. Xiao,Vygodskaya, N., 2008. Modeling carbon dynamics in two adjacent spruce forests with different soil conditions in Russia. Biogeosciences, 5(4): 969-980.

Knohl, A., Schulze, E.-D., Kolle, O.,Buchmann, N., 2003. Large carbon uptake by an unmanaged 250-year-old deciduous forest in Central Germany. Agricultural and Forest Meteorology, 118(3-4): 151-167.

Loubet, B., Laville, P., Lehuger, S., Larmanou, E., Fléchard, C., Mascher, N., Genermont, S., Roche, R., Ferrara, R.M., Stella, P., Personne, E., Durand, B., Decuq, C., Flura, D., Masson, S., Fanucci, O., Rampon, J.-N., Siemens, J., Kindler, R., Gabrielle, B., Schrumpf, M.,Cellier, P., 2011. Carbon, nitrogen and Greenhouse gases budgets over a four years crop rotation in northern France. Plant Soil, 343(1-2): 109-137.

Ma, S., Baldocchi, D.D., Xu, L.,Hehn, T., 2007. Inter-annual variability in carbon dioxide exchange of an oak/grass savanna and open grassland in California. Agricultural and Forest Meteorology, 147(3-4): 157-171.

Marcolla, B., Cescatti, A., Manca, G., Zorer, R., Cavagna, M., Fiora, A., Gianelle, D., Rodeghiero, M., Sottocornola, M.,Zampedri, R., 2011. Climatic controls and ecosystem responses drive the inter-annual variability of the net ecosystem exchange of an alpine meadow. Agricultural and forest meteorology, 151(9): 1233-1243.

Matamala, R., Jastrow, J.D., Miller, R.M.,Garten, C., 2008. Temporal changes in C and N stocks of restored prairie: implications for C sequestration strategies. Ecol. Appl., 18(6): 1470-1488.

Montagnani, L., Manca, G., Canepa, E., Georgieva, E., Acosta, M., Feigenwinter, C., Janous, D., Kerschbaumer, G., Lindroth, A., Minach, L., Minerbi, S., Mölder, M., Pavelka, M., Seufert, G., Zeri, M.,Ziegler, W., 2009. A new mass conservation approach to the study of CO2advection in an alpine forest. JGR, 114(D7).

Moureaux, C., Debacq, A., Bodson, B., Heinesch, B.,Aubinet, M., 2006. Annual net ecosystem carbon exchange by a sugar beet crop. Agricultural and Forest Meteorology, 139(1-2): 25-39.

Noormets, A., McNulty, S.G., DeForest, J.L., Sun, G., Li, Q.,Chen, J., 2008. Drought during canopy development has lasting effect on annual carbon balance in a deciduous temperate forest. New Phytol., 179(3): 818-828.

Paul Berbigier, Jean-Marc Bonnefond,Mellmann, P., 2001. CO2 and water vapour fluxes for 2 years above Euroflux forest site. Agricultural and Forest Meteorology, 108(3): 183-197.

Peichl, M., Brodeur, J.J., Khomik, M.,Arain, M.A., 2010. Biometric and eddy-covariance based estimates of carbon fluxes in an age-sequence of temperate pine forests. Agricultural and Forest Meteorology, 150(7-8): 952-965.

R. Holzinger, A. Lee, M. McKay,Goldstein, A.H., 2006. Seasonal variability of monoterpene emission factors for a Ponderosa pine plantation in California. Atmospheric Chemistry and physics 6(5): 1267-1274.

R. K. MONSON, A.A . TURN I P S E ED, J . P .S PARK S, P .C.HAR L E Y, L . E .S COT T DENTON, K. SPARKS,HUXMAN, T.E., 2002. Carbon sequestration in a high‐elevation, subalpine forest. Global Change Biol., 8(5): 459-478.

R. VALENTINI, P. De ANGELIS, G. MATTEUCCI, R. MONACO, S. DORE,MUCNOZZA, G.E.S., 1996. Seasonal net carbon dioxide exchange of a beech forest with the atmosphere. Global Change Biol., 2(3): 199-207.

Scott, R., 2016. AmeriFlux US-Wkg Walnut Gulch Kendall Grasslands, Lawrence Berkeley National Lab.(LBNL), Berkeley, CA (United States ….

Scott, R.L., Biederman, J.A., Hamerlynck, E.P.,Barron‐Gafford, G.A., 2015. The carbon balance pivot point of southwestern US semiarid ecosystems: Insights from the 21st century drought. Journal of Geophysical Research: Biogeosciences, 120(12): 2612-2624.

Stoy, P.C., Trowbridge, A.M.,Bauerle, W.L., 2014. Controls on seasonal patterns of maximum ecosystem carbon uptake and canopy-scale photosynthetic light response: contributions from both temperature and photoperiod. Photosynth Res, 119(1-2): 49-64.

Tedeschi, V., Rey, A.N.A., Manca, G., Valentini, R., Jarvis, P.G.,Borghetti, M., 2006. Soil respiration in a Mediterranean oak forest at different developmental stages after coppicing. Global Change Biol., 12(1): 110-121.

Thum, T., Aalto, T., Laurila, T., Aurela, M., Kolari, P.,Hari, P., 2007. Parametrization of two photosynthesis models at the canopy scale in a northern boreal Scots pine forest. Tellus B: Chemical and Physical Meteorology, 59(5): 874-890.

Vesala, T., Suni, T., Rannik, Ü., Keronen, P., Markkanen, T., Sevanto, S., Grönholm, T., Smolander, S., Kulmala, M., Ilvesniemi, H., Ojansuu, R., Uotila, A., Levula, J., Mäkelä, A., Pumpanen, J., Kolari, P., Kulmala, L., Altimir, N., Berninger, F., Nikinmaa, E.,Hari, P., 2005. Effect of thinning on surface fluxes in a boreal forest. GBioC, 19(2): n/a-n/a.

Vickers, D., Thomas, C.,Law, B.E., 2009. Random and systematic CO2 flux sampling errors for tower measurements over forests in the convective boundary layer. Agricultural and Forest Meteorology, 149(1): 73-83.

Vitale, L., Di Tommasi, P., D’Urso, G.,Magliulo, V., 2016. The response of ecosystem carbon fluxes to LAI and environmental drivers in a maize crop grown in two contrasting seasons. IJBm, 60(3): 411-420.

Wohlfahrt, G., Hammerle, A., Haslwanter, A., Bahn, M., Tappeiner, U.,Cernusca, A., 2008. Seasonal and inter-annual variability of the net ecosystem CO2 exchange of a temperate mountain grassland: effects of climate and management. J Geophys Res Atmos, 113(D8).

Zielis, S., Etzold, S., Zweifel, R., Eugster, W., Haeni, M.,Buchmann, N., 2014. NEP of a Swiss subalpine forest is significantly driven not only by current but also by previous year's weather. Biogeosciences, 11(6): 1627-1635.

Zimmermann, F., Plessow, K., Queck, R., Bernhofer, C.,Matschullat, J., 2006. Atmospheric N- and S-fluxes to a spruce forest—Comparison of inferential modelling and the throughfall method. Atmos. Environ., 40(25): 4782-4796.

**Table S2.** Trends of EWUE, IWUE and uWUE for all DBF, ENF, GRA and CRO sites.

| Site ID | PFT | NOY | EWUE | | IWUE | | uWUE | |
| --- | --- | --- | --- | --- | --- | --- | --- | --- |
|  |  |  | slope | *p*-value | slope | *p*-value | slope | *p*-value |
| DE-Hai | DBF | 10 | **-3.83*** | **0.05** | -1.43 | 0.37 | -2.37 | 0.11 |
| DE-Lnf | DBF | 8 | -1.21 | 0.71 | -0.60 | 0.90 | -0.89 | 0.90 |
| IT-Col | DBF | 10 | -0.03 | 0.86 | -2.14 | 0.37 | -0.66 | 0.72 |
| IT-Ro2 | DBF | 9 | 1.42 | 0.60 | 5.84 | 0.12 | 6.28 | 0.08 |
| US-MMS | DBF | 16 | 0.81 | 0.30 | 1.64 | 0.10 | 1.22 | 0.10 |
| US-Oho | DBF | 10 | **-1.59*** | **0.03** | -1.48 | 0.15 | -1.62 | 0.15 |
| US-WCr | DBF | 12 | 3.53 | 0.19 | **4.82*** | **0.02** | **3.89*** | **0.05** |
| CA-Qfo | ENF | 7 | -1.21 | 0.37 | -0.92 | 0.76 | -1.24 | 0.55 |
| CA-TP1 | ENF | 7 | **9.89*** | **0.04** | 6.09 | 0.55 | 8.02 | 0.13 |
| CA-TP3 | ENF | 7 | 3.44 | 0.07 | 0.38 | 0.76 | 1.86 | 0.37 |
| CA-TP4 | ENF | 7 | 3.30 | 0.55 | **9.71*** | **0.02** | **4.63**** | **0.01** |
| CH-Dav | ENF | 8 | 3.33 | 0.39 | 2.12 | 0.27 | 2.21 | 0.17 |
| DE-Obe | ENF | 6 | 4.72 | 0.13 | 3.29 | 0.26 | 4.04 | 0.06 |
| DE-Tha | ENF | 18 | **1.92**** | **0.01** | **2.34***** | **0.00** | **2.02***** | **0.00** |
| FI-Hyy | ENF | 18 | -0.77 | 0.29 | 0.15 | 0.88 | -0.28 | 0.54 |
| FI-Sod | ENF | 7 | 2.31 | 0.13 | 5.36 | 0.13 | 3.50 | 0.23 |
| FR-LBr | ENF | 5 | 1.89 | 1.00 | -15.27 | 0.31 | -8.15 | 0.31 |
| IT-Lav | ENF | 11 | 0.19 | 0.76 | 1.41 | 0.28 | 0.42 | 0.64 |
| IT-SRo | ENF | 11 | -1.29 | 0.76 | 1.75 | 0.35 | 0.94 | 0.88 |
| NL-Loo | ENF | 15 | -0.09 | 0.92 | -2.57 | 0.11 | -1.76 | 0.11 |
| RU-Fyo | ENF | 6 | -5.54 | 0.45 | -6.13 | 0.26 | -8.07 | 0.26 |
| US-Blo | ENF | 10 | **8.41*** | **0.03** | 6.78 | 0.07 | 7.79 | 0.11 |
| US-GLE | ENF | 6 | 0.34 | 0.45 | -2.24 | 1.00 | 0.36 | 1.00 |
| US-Me2 | ENF | 13 | -0.50 | 0.58 | -0.73 | 0.76 | -0.13 | 1.00 |
| US-NR1 | ENF | 9 | -0.94 | 0.25 | -1.21 | 0.47 | -0.87 | 0.60 |
| AT-Neu | GRA | 11 | **5.49***** | **0.00** | 2.88 | 0.28 | **4.08*** | **0.02** |
| AU-DaP | GRA | 5 | -10.53 | 0.22 | -15.64 | 0.46 | -14.15 | 0.22 |
| AU-Stp | GRA | 5 | 16.44 | 0.09 | 10.18 | 0.22 | 11.85 | 0.09 |
| CH-Cha | GRA | 10 | -2.52 | 0.72 | **4.14*** | **0.02** | 0.21 | 0.86 |
| CH-Fru | GRA | 10 | 0.13 | 1.00 | 0.75 | 0.59 | -0.94 | 0.59 |
| IT-MBo | GRA | 10 | -0.95 | 0.28 | -0.78 | 0.59 | -1.29 | 0.59 |
| IT-Tor | GRA | 5 | -4.07 | 0.09 | 0.55 | 1.00 | -1.30 | 0.46 |
| US-IB2 | GRA | 7 | -1.33 | 0.37 | -3.74 | 0.37 | -2.83 | 0.07 |
| US-SRG | GRA | 7 | 0.90 | 1.00 | 2.05 | 0.55 | 0.55 | 0.55 |
| US-Var | GRA | 14 | -0.07 | 1.00 | 1.06 | 0.44 | 0.78 | 0.51 |
| US-Wkg | GRA | 11 | -0.37 | 1.00 | -0.89 | 0.76 | -0.76 | 0.76 |
| BE-Lon | CRO | 10 | 2.94 | 0.37 | 2.46 | 0.72 | 3.51 | 0.72 |
| DE-Geb | CRO | 10 | 1.12 | 0.86 | 3.56 | 0.28 | 3.69 | 0.47 |
| DE-Kli | CRO | 10 | -5.52 | 0.15 | **-4.45*** | **0.05** | -4.93 | 0.11 |
| FR-Gri | CRO | 7 | 5.55 | 0.55 | 3.00 | 0.76 | 4.36 | 0.55 |
| IT-BCi | CRO | 9 | **-10.63***** | **0.00** | **-13.88***** | **0.00** | **-12.73***** | **0.00** |
| US-ARM | CRO | 10 | 3.64 | 0.07 | 1.54 | 0.59 | 1.56 | 1.00 |

Note: The *p-*value is derived from the Mann-Kendall trend test for each site. The slope (% yr^-1^) was calculated using Sen’s slope estimator. *indicates significance at the 0.05 level; **indicates significance at the 0.01 level; ***indicates significance at the 0.001 level.

**Table S3.** EWUE, IWUE and uWUE trends (% yr^-1^) of DBF, ENF, GRA and CRO obtained by Sen's slope estimator method.

| PFT | NOY | EWUE | | IWUE | | uWUE | |
| --- | --- | --- | --- | --- | --- | --- | --- |
|  |  | slope | *p*-value | slope | *p*-value | slope | *p*-value |
| DBF | 16 | 0.25 | 0.82 | 1.80 | 0.12 | 0.93 | 0.08 |
| ENF | 18 | **0.53** | **0.05** | 0.29 | 0.71 | 0.54 | 0.17 |
| GRA | 14 | **-2.51** | **0.05** | 0.94 | 0.19 | -0.98 | 0.23 |
| CRO | 14 | 2.71 | 0.32 | 0.69 | 0.91 | 1.19 | 0.19 |

Note: The *p-*value is derived from the Mann-Kendall trend test for each site. Black bold font indicate that the trends are significant.

**Table S4.** EWUE, IWUE and uWUE trends (% yr^-1^) of DBF, ENF, GRA and CRO obtained by Bootstrap method. Cilow and Cihigh represent 95% confidence intervals for trends.

| Variables | PFT | Slope | Cilow | Cihigh |
| --- | --- | --- | --- | --- |
| EWUE | DBF | -0.12 | -1.28 | 0.65 |
| IWUE | DBF | 0.59 | -1.13 | 2.92 |
| uWUE | DBF | 0.39 | -1.14 | 2.07 |
| EWUE | ENF | **1.57** | **0.72** | **3.58** |
| IWUE | ENF | 0.99 | -0.46 | 2.49 |
| uWUE | ENF | 0.96 | -0.52 | 2.39 |
| EWUE | GRA | -0.31 | -1.71 | 1.32 |
| IWUE | GRA | 0.67 | -1.06 | 1.81 |
| uWUE | GRA | -0.17 | -1.23 | 1.03 |
| EWUE | CRO | 0.55 | -5.52 | 2.84 |
| IWUE | CRO | 0.64 | -2.95 | 2.50 |
| uWUE | CRO | 0.96 | -4.93 | 3.11 |

Note: Black bold font indicate that the trends are significant.

**Table S5.** EWUE, IWUE and uWUE trends (% yr^-1^) of DBF, ENF, GRA and CRO obtained by Bootstrap method. Cilow and Cihigh represent 90% confidence intervals for trends.

| Variables | PFT | Slope | Cilow | Cihigh |
| --- | --- | --- | --- | --- |
| EWUE | DBF | -0.12 | -1.13 | 0.53 |
| IWUE | DBF | 0.59 | -0.86 | 2.46 |
| uWUE | DBF | 0.39 | -1.04 | 1.72 |
| EWUE | ENF | **1.57** | **0.88** | **3.39** |
| IWUE | ENF | 0.99 | -0.18 | 2.30 |
| uWUE | ENF | 0.96 | -0.26 | 2.17 |
| EWUE | GRA | -0.31 | -1.51 | 0.92 |
| IWUE | GRA | 0.67 | -0.75 | 1.62 |
| uWUE | GRA | -0.17 | -1.11 | 0.79 |
| EWUE | CRO | 0.55 | -3.86 | 2.66 |
| IWUE | CRO | 0.64 | -2.95 | 2.46 |
| uWUE | CRO | 0.96 | -3.31 | 3.07 |

Note: Black bold font indicate that the trends are significant.

**Table S6.** Normalized coefficients from PLSR between the annual anomalies of EWUE/IWUE/uWUE and meteorological variables for Significant and Non-significant sites.

| Variables | PFT | EWUE | | IWUE | | uWUE | |
| --- | --- | --- | --- | --- | --- | --- | --- |
|  |  | coefficient | *p*-value | coefficient | *p*-value | coefficient | *p*-value |
| TA | Significant | -0.099 | 0.62 | -0.095 | 0.595 | -0.102 | 0.608 |
| VPD | Significant | **-0.353+** | **0.062** | 0.271 | 0.119 | -0.042 | 0.829 |
| Ca | Significant | **0.161+** | **0.083** | **0.159*** | **0.03** | **0.17+** | **0.053** |
| SR | Significant | 0.141 | 0.549 | 0.251 | 0.203 | 0.225 | 0.32 |
| SWC | Significant | 0.048 | 0.577 | -0.037 | 0.646 | 0.002 | 0.977 |
| TA | Non-significant | 0.003 | 0.964 | 0.15 | 0.198 | 0.096 | 0.175 |
| VPD | Non-significant | -0.139 | 0.202 | **0.435***** | **0** | **0.179+** | **0.052** |
| Ca | Non-significant | -0.001 | 0.984 | 0.026 | 0.803 | 0.013 | 0.859 |
| SR | Non-significant | -0.075 | 0.572 | 0.027 | 0.782 | -0.006 | 0.96 |
| SWC | Non-significant | -0.095 | 0.133 | -0.016 | 0.806 | -0.06 | 0.346 |

Note: The meteorological variables include air temperature (TA), vapor pressure deficit (VPD), atmospheric CO_2_ concentration (Ca), shortwave radiation (SR), soil water content (SWC). + indicates significance at the 0.1 level; *indicates significance at the 0.05 level; **indicates significance at the 0.01 level; ***indicates significance at the 0.001 level.

**Table S7.** Normalized coefficients from PLSR between the annual anomalies of EWUE/IWUE/uWUE and meteorological variables for ALLs, DBFs, ENFs, GRAs and CROs.

| Variables | PFT | EWUE | | IWUE | | uWUE | |
| --- | --- | --- | --- | --- | --- | --- | --- |
|  |  | coefficient | *p*-value | coefficient | *p*-value | coefficient | *p*-value |
| TA | ALL | 0.013 | 0.862 | 0.071 | 0.314 | 0.056 | 0.44 |
| VPD | ALL | **-0.198*** | **0.04** | **0.42***** | **0** | 0.138 | 0.126 |
| Ca | ALL | 0.052 | 0.452 | 0.077 | 0.13 | 0.064 | 0.29 |
| SR | ALL | -0.044 | 0.56 | 0.086 | 0.282 | 0.034 | 0.635 |
| SWC | ALL | -0.073 | 0.141 | -0.02 | 0.713 | -0.05 | 0.327 |
| TA | DBF | 0.29 | 0.239 | **0.191*** | **0.028** | 0.282 | 0.105 |
| VPD | DBF | -0.411 | 0.121 | **0.48***** | **0.001** | 0.084 | 0.7 |
| Ca | DBF | -0.071 | 0.549 | -0.095 | 0.414 | -0.095 | 0.395 |
| SR | DBF | 0.114 | 0.596 | **0.203**** | **0.006** | 0.194 | 0.191 |
| SWC | DBF | -0.028 | 0.869 | -0.034 | 0.778 | -0.042 | 0.714 |
| TA | ENF | -0.113 | 0.423 | -0.094 | 0.479 | -0.11 | 0.438 |
| VPD | ENF | 0.055 | 0.747 | **0.69***** | **0** | **0.433*** | **0.011** |
| Ca | ENF | **0.159+** | **0.065** | 0.076 | 0.271 | 0.108 | 0.183 |
| SR | ENF | -0.128 | 0.127 | -0.042 | 0.568 | -0.078 | 0.267 |
| SWC | ENF | -0.001 | 0.995 | -0.039 | 0.594 | -0.029 | 0.733 |
| TA | GRA | -0.077 | 0.521 | **0.196+** | **0.063** | 0.077 | 0.529 |
| VPD | GRA | **-0.195**** | **0.007** | **0.251**** | **0.002** | 0.048 | 0.55 |
| Ca | GRA | 0.176 | 0.11 | 0.139 | 0.224 | 0.176 | 0.166 |
| SR | GRA | **-0.365**** | **0.004** | 0.037 | 0.625 | **-0.17+** | **0.079** |
| SWC | GRA | -0.168 | 0.261 | **-0.191+** | **0.084** | -0.202 | 0.161 |
| TA | CRO | -0.049 | 0.76 | 0.15 | 0.276 | 0.06 | 0.691 |
| VPD | CRO | -0.182 | 0.159 | 0.144 | 0.226 | -0.008 | 0.949 |
| Ca | CRO | -0.047 | 0.734 | -0.066 | 0.506 | -0.06 | 0.616 |
| SR | CRO | 0.257 | 0.192 | 0.249 | 0.103 | 0.263 | 0.125 |
| SWC | CRO | 0.032 | 0.788 | 0.017 | 0.856 | 0.025 | 0.813 |

Note: The meteorological variables include air temperature (TA), vapor pressure deficit (VPD), atmospheric CO_2_ concentration (Ca), shortwave radiation (SR), soil water content (SWC). + indicates significance at the 0.1 level; *indicates significance at the 0.05 level; **indicates significance at the 0.01 level; ***indicates significance at the 0.001 level.

**Table S8.** The relative importance of meteorological variables to dependent variables (EWUE, IWUE, uWUE) under different sites (Significant, Non-significant). Meteorological variables include air temperature (TA), vapor pressure deficit (VPD), atmospheric CO_2_ concentration (Ca), shortwave radiation (SR), soil water content (SWC).

| PFT | Variables | EWUE | IWUE | uWUE |
| --- | --- | --- | --- | --- |
| Significant | TA | 9.93% | 3.00% | 7.95% |
| Significant | VPD | 63.84% | 47.58% | 3.37% |
| Significant | Ca | 17.36% | 11.52% | 44.63% |
| Significant | SR | 6.38% | 37.42% | 43.55% |
| Significant | SWC | 2.49% | 0.49% | 0.50% |
| Non-significant | TA | 7.06% | 24.20% | 30.37% |
| Non-significant | VPD | 49.89% | 63.32% | 52.06% |
| Non-significant | Ca | 0.20% | 0.15% | 0.16% |
| Non-significant | SR | 31.61% | 7.43% | 4.40% |
| Non-significant | SWC | 11.24% | 4.90% | 13.02% |

**Table S9.** The relative importance of meteorological variables to dependent variables (EWUE, IWUE, uWUE) under different biomes (ALL, DBF, ENF, GRA, CRO). Meteorological variables include air temperature (TA), vapor pressure deficit (VPD), atmospheric CO_2_ concentration (Ca), shortwave radiation (SR), soil water content (SWC).

| PFT | Variables | EWUE | IWUE | uWUE |
| --- | --- | --- | --- | --- |
| ALL | TA | 8.06% | 17.00% | 20.84% |
| ALL | VPD | 65.38% | 65.45% | 47.80% |
| ALL | Ca | 8.51% | 1.51% | 8.39% |
| ALL | SR | 14.67% | 12.01% | 11.55% |
| ALL | SWC | 3.38% | 4.02% | 11.41% |
| DBF | TA | 34.79% | 25.77% | 47.66% |
| DBF | VPD | 52.64% | 47.85% | 18.36% |
| DBF | Ca | 3.39% | 0.40% | 0.89% |
| DBF | SR | 6.65% | 19.39% | 26.70% |
| DBF | SWC | 2.54% | 6.59% | 6.39% |
| ENF | TA | 18.84% | 11.86% | 8.84% |
| ENF | VPD | 3.86% | 77.46% | 72.83% |
| ENF | Ca | 47.52% | 2.93% | 11.52% |
| ENF | SR | 29.49% | 4.67% | 3.11% |
| ENF | SWC | 0.29% | 3.08% | 3.70% |
| GRA | TA | 6.88% | 16.90% | 2.95% |
| GRA | VPD | 30.49% | 58.28% | 11.29% |
| GRA | Ca | 4.39% | 8.01% | 27.56% |
| GRA | SR | 51.89% | 8.83% | 30.60% |
| GRA | SWC | 6.36% | 7.98% | 27.60% |
| CRO | TA | 2.17% | 20.53% | 8.46% |
| CRO | VPD | 37.17% | 23.71% | 0.86% |
| CRO | Ca | 1.62% | 1.57% | 3.08% |
| CRO | SR | 58.19% | 53.82% | 86.92% |
| CRO | SWC | 0.85% | 0.38% | 0.67% |

**Table S10.** Correlation coefficients between the independent variables for DBFs, ENFs, GRAs and CROs.

|  | TA | VPD | Ca | SR | SWC |
| --- | --- | --- | --- | --- | --- |
| DBF.TA | 1 | **0.58***** | 0.07 | 0.05 | -0.13 |
| DBF.VPD |  | 1 | **0.25*** | **0.3**** | **-0.27*** |
| DBF.Ca |  |  | 1 | 0.22 | 0.05 |
| DBF.SR |  |  |  | 1 | **0.23*** |
| DBF.SWC |  |  |  |  | 1 |
| ENF.TA | 1 | **0.60***** | 0.14 | **0.34***** | **-0.28***** |
| ENF.VPD |  | 1 | 0.13 | **0.52***** | **-0.28***** |
| ENF.Ca |  |  | 1 | -0.01 | 0.07 |
| ENF.SR |  |  |  | 1 | -0.11 |
| ENF.SWC |  |  |  |  | 1 |
| GRA.TA | 1 | **0.60***** | -0.11 | 0.20 | -0.11 |
| GRA.VPD |  | 1 | 0.01 | **0.40***** | **-0.29**** |
| GRA.Ca |  |  | 1 | -0.01 | 0.08 |
| GRA.SR |  |  |  | 1 | 0.02 |
| GRA.SWC |  |  |  |  | 1 |
| CRO.TA | 1 | **0.65***** | 0 | **0.60***** | **-0.25** |
| CRO.VPD |  | 1 | -0.05 | **0.32*** | **-0.35**** |
| CRO.Ca |  |  | 1 | 0.04 | -0.04 |
| CRO.SR |  |  |  | 1 | -0.03 |
| CRO.SWC |  |  |  |  | 1 |

Note: All the independent variables were transformed into the z-scores of the relative annual changes for the calculation. A high correlation coefficient implies a collinearity between the independent variables. *indicates significance at the 0.05 level; **indicates significance at the 0.01 level; ***indicates significance at the 0.001 level.
